# Epigenetic regulation in female reproduction: the impact of m6A on maternal-fetal health

**DOI:** 10.1038/s41420-025-02324-z

**Published:** 2025-02-04

**Authors:** Peipei Li, Yumeng Lin, Hongyun Ma, Jiao Zhang, Qiaorui Zhang, Ruihua Yan, Yang Fan

**Affiliations:** 1 Department of Obstetrics and Gynecology, People’s Hospital of Ningxia Hui Autonomous Region, Yinchuan, Ningxia, China; 2 Health Management Center, Nanjing Tongren Hospital, School of Medicine, Southeast University, Nanjing, China

**Keywords:** Epigenetics, RNA modification

## Abstract

With the development of public health, female diseases have become the focus of current concern. The unique reproductive anatomy of women leads to the development of gynecological diseases gradually become an important part of the socio-economic burden. Epigenetics plays an irreplaceable role in gynecologic diseases. As an important mRNA modification, m6A is involved in the maturation of ovum cells and maternal-fetal microenvironment. At present, researchers have found that m6A is involved in the regulation of gestational diabetes and other reproductive system diseases, but the specific mechanism is not clear. In this manuscript, we summarize the components of m6A, the biological function of m6A, the progression of m6A in the maternal-fetal microenvironment and a variety of gynecological diseases as well as the progression of targeted m6A treatment-related diseases, providing a new perspective for clinical treatment-related diseases.

## Facts

- m6A Modification in Female Reproduction: The role of N6-methyladenosine (m6A) in female reproduction is an emerging field, with evidence suggesting its involvement in oocyte maturation, embryonic development, and placental function, which are crucial for maternal-fetal health.
- Epigenetic Regulation in Gynecological Diseases: m6A modification has been implicated in various gynecological diseases, including cervical cancer, ovarian cancer, endometrial cancer, and choriocarcinoma, potentially affecting their progression and treatment outcomes.
- Technological Advances in m6A Detection: Recent developments in high-throughput sequencing and other detection technologies have expanded our ability to study m6A modifications at single-base resolution, offering new opportunities for understanding the mechanisms of gene regulation.
- m6A’s Impact on Immune Cells: The influence of m6A on immune cells within the maternal-fetal microenvironment, such as dendritic cells, natural killer cells, and macrophages, suggests a significant role in modulating immune tolerance and responses during pregnancy.
- Clinical Implications and Challenges: The potential of m6A as a biomarker and therapeutic target and biomarker in gynecological diseases is evident, but the complexity of its regulatory mechanisms and the need for a comprehensive understanding of its role in different pathological contexts present challenges for clinical application.

## Open Questions

- How does m6A modification specifically regulate the balance between immune tolerance and response in the maternal-fetal microenvironment, and what are the long-term implications for both maternal and fetal health?
- What are the precise molecular mechanisms by which m6A modification influences the progression of various gynecological cancers, and how can this knowledge be leveraged to develop targeted therapies?
- Given the recent advancements in m6A detection technologies, what new insights can be gained about the role of m6A in non-malignant gynecological conditions such as endometriosis and polycystic ovary syndrome?
- Can m6A modification patterns serve as reliable biomarkers for the diagnosis, prognosis, and treatment response monitoring in gynecological diseases, and what validation is needed to implement them in clinical practice?
- How do environmental factors and lifestyle choices impact m6A methylation patterns in the context of female reproduction and gynecological health, and what preventive strategies can be developed based on this knowledge?

## Introduction

Epitranscriptomics refers to the dynamic and reversible process of chemically changing several kinds of RNA molecules, including mRNAs and non-coding RNAs. These modifications play a critical role in governing RNA functionality and determining its fate, as well as influencing the gene expression process [1, 2]. The discovery of over 170 RNA alterations in various organisms has led to an increasing number of studies being conducted to elucidate their precise pathways in the development of disease [2]. The primary epitranscriptomic modifications consist of N6-methyladenosine (m6A) [3], N1-methyladenosine (m1A) [4], 5-methylcytidine (m5C) [5], and pseudouridine (Ψ) [6]. Out of all the chemical modifications in eukaryotic messenger RNAs, m6A is regarded as the most prevalent [3]. The particular mechanism of m6A modification in RNA involves the insertion of a methyl group (CH3) at the N6 position of adenine A in the RNA molecule. As an essential element of the epigenetic, m6A modification dynamically regulates multiple aspects of gene expression, containing RNA splicing, stability, translation, and degradation. Furthermore, m6A modification interacts closely with other epigenetic mechanisms, including DNA methylation and histone alterations, collectively contributing to the precise regulation of gene expression. DNA methylation is typically correlated to gene silencing, while m6A modification and histone modifications primarily center on post-transcriptional regulation. The coordinated interplay of these three modifications plays an essential role in biological processes, regulating cellular fate, immune responses, and the development of various diseases [7, 8].

Women’s reproductive health is a crucial component of societal progress [9]. Currently, due to the fast-paced socio-economic progress, there is a heightened global focus on reproductive health. However, a significant number of women continue to experience various reproductive health issues, including gynecological cancer, pregnancy-related ailments, infertility, gynecological chronic diseases, and more. These conditions can have detrimental effects on women’s physical health, family life, and economic well-being, and in severe cases, they can even be life-threatening [10, 11]. Hence, comprehending the mechanism and target of associated diseases is crucial for resolving issues pertaining to clinical treatment.

**Fig. 1:**
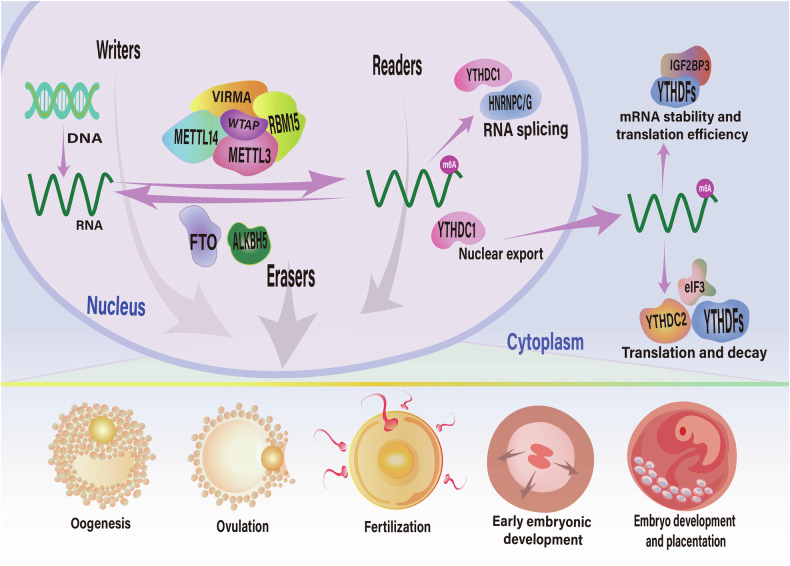
The composition, biological functions, and potential roles of m6A modifications in the female reproductive system. m6A writers (METTL3, METTL14, WTAP, RBM15/15B, VIRMA), m6A erasers (ALKBH5 and FTO), and m6A readers (YTH family protein and hnRNPs protein) are the primary regulators of the m6A modification. m6A methylation modification is involved in the regulation of alternative splicing, nuclear export, degradation, translation, and RNA stability. In the process of female reproduction, m6A RNA modification is also essential for oogenesis, ovulation, and embryonic development.

The study of epigenomics has steadily confirmed the connection between m6A modification and the physiological and pathological processes in the female reproductive system [12] (Fig. 1). As an illustration, it has been elucidated that m6A modification has a substantial influence on follicular development and oocyte growth. Based on RNA-seq and Me RIP-seq results, researchers discovered that Itsn2 is targeted for m6A modification by METTL3, thereby enhancing its stability and then affecting the oocyte meiotic process [12, 13]. Itsn2 is a critical adapter protein that has a significant role in regulating oocyte meiotic resumption [14]. The presence of m6A modification in ovarian tissue of individuals diagnosed with polycystic ovary syndrome (PCOS) is dysregulated, which disrupts follicular development and growth, ultimately leading to ovarian dysfunction and the pathogenesis of PCOS. This process involves the alteration of key signaling pathways, including PI3K/Akt and mTOR, which play critical roles in regulating ovarian function and folliculogenesis [15]. m6A regulators appear to be involved in reproductive aging. Sun et al. evaluated the levels of expression of FTO and m6A methylation levels within various germ cells and found that FTO expression gradually decreased but m6A showed an increasing trend during the aging process [16]. In order to ascertain the precise mechanism at play, additional research is required. Furthermore, pertinent investigations have suggested that m6A additionally contributes to mechanisms associated with gynecological cancer [17–19]. m6A modification regulates tumor growth and dissemination by regulating the expression levels of oncogenes and tumor suppressor genes. Consequently, clinical research may leverage m6A modification as a diagnostic and prognostic biomarker for various diseases affecting the female reproductive system, enabling personalized diagnosis and treatment through the identification of potential therapeutic targets. m6A modification is increasingly recognized for its significant role in female reproductive health. However, further investigation is essential to more comprehensively understand the correlation between m6A modification and female reproductive system function, as well as the deeper mechanisms involved.

This manuscript presents an exhaustive review of m6A regulators and their biological function-related mechanisms, as well as the progress made in detecting m6A. Our emphasis is on the pivotal role of m6A modification in shaping and regulating the maternal-fetal immune microenvironment, as well as influencing the treatment and prognosis of obstetrics and gynecology-related diseases. We provide obstetricians and gynecologists with a new perspective to understand associated diseases more thoroughly, which will improve their future comprehension of these conditions.

## m6A modification mechanism and detection method

### m6A modification

#### m6A writer

The m6A writers refer to a group of enzymes or complexes that are mainly tasked with the methylation of the target. METTL3, METTL14, and Wilms’ tumor-associated protein (WTAP) will be the subject of this section [8]. For instance, the initial two are regulated by WTAP. Moreover, ancillary subunits, including RNA-binding motif protein 15 (RBM15), zinc finger CCCH domain protein 13 (ZC3H13), and vir-like m6A methyltransferase-associated subunit (VIRMA, also known as KIAA1429), are involved in the mechanism [20–22]. These methyltransferases constitute the methyltransferase complex (MTC). The MTC performs its function by introducing methyl groups to certain adenosine residues in RNA, resulting in m6A modifications [8].

METTL3 is the initial m6A writer identified as possessing the only catalytic subunit that helps methyl transfer happen when it binds to S-Adenosyl Methionine (SAM), a cellular methyl donor. METTL3 is mainly localized in the nucleus, although it is also existing in the cytoplasm [2, 8, 23]. It is pivotal in controlling the cell cycle through the modulation of key gene expression required for cell division. Within the tumor microenvironment, METTL3 contributes to immune evasion and tumor angiogenesis by regulating the secretion of immune cells and cytokines. Additionally, METTL3 has been demonstrated to facilitate the initiation and progression of gynecologic cancers through the modulation of several critical signaling pathways such as the PI3K/Akt and Wnt/β-catenin [17]. METTL14 possesses a SAM-dependent methyltransferase domain and an RNA-binding domain, which is structurally similar to METTL3 [24]. METTL14 indirectly contributes to the catalytic process by binding to METTL3 and stabilizing its conformation [23]. WTAP is a crucial member of the MTC, which regulates RNA m6A modification by binding to RNA molecules and interacting with proteins. Despite the absence of methyltransferase activity, WTAP facilitates the localization of the METTL3-METTL14 complex to nuclear speckles, thereby enhancing its catalytic activity [20, 25]. The RBM15/15B and KIAA1429 attach to the MTC and guide its correct localization. The former functions as an adaptor protein, attracting MTC to U-rich regions for m6A modification [21], whereas the latter mainly guides m6A modification adjacent the 3ʹ untranslated region (3ʹ UTR) and stop codon [22].

Furthermore, WTAP and ZC3H13 are predominantly localized in the nucleus, where they modulate post-transcriptional modifications of RNA. As essential regulators of m6A methylation, these factors hold potential as novel therapeutic targets for treating gynecological cancers. ZC3H13 plays a critical auxiliary function in the MTC by anchoring and mediating nuclear m6A modification through interaction with WTAP, thereby enhancing the accuracy and efficiency of m6A modification [21, 26]. METTL5 and tRNA methyltransferase activator subunit 112 (TRMT112) mainly involved in rRNA m6A modification. TRMT112 is the only m6A rRNA methyltransferase that contains a conserved domain and associates with other methyltransferases to exercise its supplementary impact [27].

The discovery of zinc finger CCHC type containing 4 (ZCCHC4) and METTL16 occurred recently [28, 29]. METTL16 contains a canonical SAM-dependent methyltransferase domain and an RNA-binding domain. The SAM binding domain supplies methyl groups, while the RNA binding domain enhances the efficiency and accuracy of m6A modification, hence enhancing the methyltransferase activity of METTL16 [29, 30]. ZCCHC4 possesses multiple CCHC-type zinc finger domains, allowing it to regulate the terminal RNA methylation modification by binding to specific RNA sequences [29, 31, 32]. Moreover, Bawankar et al. led a study on the impact of m6A in Drosophila, which indicates that a factor called HAKAI may affect the biological function of MTC by interacting with MTC subunits through its ubiquitination domain [33]. Further investigations are needed to ascertain the exact method and value of HAKAI in m6A modification.

#### m6A reader

m6A readers are a group of proteins that can recognize and bind to m6A modifications involved in multiple m6A-mediated biological processes, and they play irreplaceable roles in regulating RNA fate. The different binding modes of m6A allow us to divide m6A-binding proteins into two categories. First, RNA recognizes and binds the m6A modification directly, such as the YT521-B homology domain (YTH) family, eukaryotic translation initiation factor 3 (eIF3), and the insulin-like growth factor 2 mRNA-binding proteins (IGF2BPs) family.

The YTHDF family protein possesses a highly conserved YTH domain, which contains a hydrophobic pocket composed of amino acids and serves as the structural component responsible for recognizing m6A [34, 35]. YTHDF1 is primarily localized in the cytoplasm, where it plays a crucial role in regulating RNA translation process [36]. In gynecologic tumors, YTHDF1 facilitates cancer progression by modulating genes associated with tumor proliferation and invasion, and it may also contribute to chemotherapy resistance. YTHDF2 can recruit mRNA degradation complexes, such as the CCR4-NOT complex, to impact the stability of RNA and accelerate the decline of targeted RNA [37, 38]. YTHDF3 contributes to the regulation of the first two [38]. YTHDC1 promotes regulated splicing by binding Serine/Arginine-Rich Splicing Factor 3 (SRSF3) and Serine/Arginine-Rich Splicing Factor 10 (SRSF10) [39, 40]. YTHDC2 plays a role in m6A modification-mediated nuclear export and RNA degradation [41].

According to pertinent research findings, the four K homology (KH) domains and two RNA recognition motifs (RRMs) of IGF2BPs, which are responsible for RNA recognition and binding, have a substantial impact on cellular stress responses. IGF2BPs are mainly found in the cytoplasm, where they regulate the expression of multiple oncogenes, thereby influencing the progression of reproductive tumors, chemotherapy resistance, and tumor immunity. IGF2BPs directly recognize and bind m6A-modified RNA via their KH domain [42]. This direct binding is highly selective and efficient since it specifically identifies and attaches to the m6A modification site.IGF2BPs associated with m6A modification mainly include IGF2BP1, IGF2BP2, and IGF2BP3, which have been demonstrated to contribute to RNA stability [43, 44], translational regulation [44], metabolic regulation [45], embryonic development [46], and cancer-related mechanisms [47], respectively. EIF3 is a multi-component structure consisting of 13 subunits, which plays an essential role in controlling the process of translation. The m6A mutation enables eIF3 binding at the 5ʹUTR region, thereby facilitating translation [48].

Besides, RNA indirectly identifies and binds to m6A modifications, including the heterogeneous nuclear ribonucleoproteins (HNRNPs) family, which consists of HNRNPC, HNRNPG, and HNRNPA2B1. HNRNPs, primarily located in the nucleus but also partially present in the cytoplasm, contribute to the proliferation and migration of tumor cells by modulating the translation of genes associated with the cell cycle [49]. HNRNPs play a critical regulatory role in gynecologic cancers. Targeting HNRNPs could provide a promising therapeutic approach to inhibit the progression of these malignancies. HNRNPs indirectly recognize m6A-modified RNAs through their RRM. m6A modification can change the secondary structure of RNA, increase the binding affinity of HNRNPC to transcripts, and reflect the conversion effect of m6A [50]. HNRNPA2B1 has been demonstrated to bind to m6A-modified RNA and enhance METTL3-dependent alternative splicing processes [51]. Relevant research indicates that HNRNPC/G also participates in alternative splicing of pre-RNAs through various mechanisms, which require specific elucidation in the future [52].

Fragile X Mental Retardation Protein (FMRP) and proline-rich coiled-coil 2 A (Prrc2a) may play a role in the regulation of RNA m6A modification regulatory networks. Researchers have found that they also contribute to neurodevelopment and spermatogenesis [53, 54]. Although these proteins do not belong to the category known as classical m6A reader proteins, they have the potential to help find new ways to treat diseases that are related.

#### m6A eraser

The m6A erasers refer to a group of demethylases that are accountable for the elimination of the m6A modification. With the combined involvement of Fe^2+^ and α-ketoglutarate, m6A methylation modification can be eliminated by ALKB homolog 5 (ALKBH5) and fat mass and obesity-associated protein (FTO) [55, 56]. FTO and ALKBH5 are localized predominantly in the nucleus, where they exert demethylation activity through the core catalytic domain, most notably the DSBH domain [57], recognizing and binding m6A-modified RNA. It has a significant impact on multiple critical stages in RNA metabolism, such as alternative splicing [55], stability [43] etc. Variants in the FTO gene have been reported to be correlated with the progress of cancer [58] and metabolic disorders like obesity [59]. Moreover, ALKBH5 plays a key role in regulating gene expression and germ cells function [56]. Recent researchers have revealed the biological role of ALKBH3 in removing the m6A modification of tRNAs [60]. As technology advances, the role of ALKBH3 in RNA modification will be further illuminated through additional details.

### m6A detection technology

Early studies explored m6A modification in RNA by radiolabeling methods such as radioisotope labeling [7]. In addition, enzyme-linked immunosorbent assays (ELISA) utilize anti-m6A antibodies to detect m6A levels in RNA, mainly for quantitative analysis. Dot blot is a rapid method for detecting m6A modification in RNA samples. The specific mechanism is to provide a basis for subsequent finer quantitative analysis by spotting RNA samples on the membrane and detecting them using m6A-specific antibodies or other probes [61].

Traditional immunoassay techniques have advantages in ease of operation and rapid detection for measuring global levels of m6A modification in RNA samples. Mass spectrometry-based methods for the detection of RNA methylation, mainly including two-dimensional thin-layer chromatography (2D-TLC) and liquid chromatography tandem mass spectrometry (LC-MS/MS), are used for quantitative analysis and detection of the presence of m6A modifications [62, 63]. LC-MS/MS involves several steps. First, RNA samples are enzymatically hydrolyzed into nucleotides. Then, the m6A content is analyzed using liquid chromatography separation, followed by UV detection through mass spectrometry [64, 65]. The method is simple in its steps, but it cannot locate sequence information and carries the risk of contamination.

Researchers then created a technique called site-specific cleavage and radiolabeled linkage-assisted extraction and thin-layer chromatography (SCARLET), which combines the benefits of solid phase extraction and thin-layer chromatography to identify methylation at specific sites and make substantial progress in detecting RNA m6A modification [66]. The single-base extension and ligation-based qPCR amplification (SELECT) method is ideal for analyzing specific monomethylation patterns on target RNAs. It allows for the precise and quantitative identification of m6A modifications at the level of individual bases in transcripts that are present in low quantities [67].

High-throughput sequencing technologies have significantly enhanced the methodologies for localizing and quantifying m6A modifications. Methylated RNA Collaborative immunoprecipitation sequencing (MERIP-seq) is the first high-throughput sequencing technique based on m6A antibody immunoprecipitation applied to m6A modification detection, which is mainly suitable for m6A modification fragment detection with sizes of 100 nt to 200 nt [68]. However, the low resolution limits its potential application. m6A-seq has improved detection accuracy over MERIP-seq [69]. The m6A-seq2 method, which was recently updated, enhances the precision and reliability of m6A quantification at site, gene, and sample levels through the integration of multiple m6A immunoprecipitation methods, utilizing mixed samples and barcoding systems [70]. The m6A-LAIC-seq method, which adds a comparison step to adjacent unenriched RNA fragments, achieves quantification of m6A levels within the transcriptome range [71]. A recent study proposed a technique based on m6A modification and transcriptomic sequencing at the single-cell level. The m6A CUT &Tag (m6A-CT) and single-nucleus m6A CUT&Tag (sn-m6A-CT) methods identify m6A-modified RNAs through a CUT&Tag-based approach, offering technical support for an in-depth study of m6A modification and comprehensive transcriptome analysis [72].

Compared to MERIP-seq, photocrosslinking-assisted m6A sequencing (PA-m6A-seq) offers a higher resolution of nearly 23–30 nt. In this method, m6A antibodies and 4-thiouridine (4SU) are mixed to make antibody and m6A-modified RNA cross-linking reactions happen. This is done by shining UV365 mm ultraviolet light on them [73]. However, due to method limitations, it is only suitable for cell culture. m6A single nucleotide resolution crosslinking and immunoprecipitation (mi CLIP) and m6A crosslinking and immunoprecipitation (m6A-CLIP) are also assays for RNA m6A modification by UV254 photocrosslinking, and they achieve high-precision m6A localization at single base resolution [74–76]. Combining mi CLIP with high-throughput sequencing (mi CLIP-seq) further enhanced the detection efficiency for m6A.

Following this, researchers have developed several new methods to enhance CLIP technology, including light-activated ribonucleoside-enhanced CLIP (PAR-CLIP) [77], enhanced CLIP (eCLIP) [78], miCLIP2 combined with machine learning [79], and single-end enhanced CLIP (seCLIP-seq) [80]. These techniques compensate for the limitations of previous CLIP approaches and enable more precise and comprehensive studies of RNA-protein complexes. The m6A-cross-linked exoribonuclease sequencing (m6ACE) technology discovered in 2019 is an upgrade to the above technology, which performs accurate quantitative analysis of m6A RNA by combining photocrosslinking, exoribonuclease, and sequencing steps [81]. However, this method is plagued by exorbitant expenses and intricate analysis.

m6A-sensitive RNA-endonuclease-facilitated sequencing (m6A-REF-seq) is a method independent of antibodies, specific endonuclease-based RNA high-throughput sequencing technique that can be used to precisely localize and analyze m6A modifications in RNA [82]. MazF is an endonuclease that can find and cut ACA motifs that have been changed by non-m6A methylation. It can also find m6A sites at a single base and cut them. MazF-coupled RNA sequencing (MAZTER-seq) is also based on ACA sequence-specific cleavage in MazF-based RNA, which allows for measuring m6A levels at the single-base level [83]. Kate D. Meyer proposed a DART-seq method to quantitatively measure genome-wide RNA m6A modification using apolipoprotein B mRNA editing enzyme catalytic subunit 1 (APOBEC1) chimeric m6A-bound YTH domains to deaminate adjacent sites m6A in 2019 [84]. The technique is also antibody-independent and shows a high level of specificity. In 2022, the researchers upgraded the aforementioned methods to detect m6A modification at single-cell resolution [85].

N6-methyladenine labeling sequencing (m6A-label-seq) and m6A-selective allyl chemical labeling (m6A-SEAL) are two new chemical labeling methods that combine high-throughput sequencing methods for finding m6A without antibodies. The former relies on FTO enzymatic activity to transform m6A into N6-hydroxymethyladenosine (hm6A), which then transforms into N6-dithioglycolylmethyladenosine (dm6A) using biotin-labeled dm6A, subsequently undergoing enrichment and additional sequencing analysis [86]. The latter employs a specific chemical reaction to react with the m6A site, introduces an allyl group, and transforms the m6A-modified RNA site into an allyl modification. This process allows for its enrichment and further analysis by antibodies, ultimately determining the distribution and levels of the m6A modification [87].

In 2022, selective allyl chemical marker sequencing (m6A-SAC-seq) specifically labeled the m6A locus using dimethyl transferase MjDim1, and after a series of chemical reactions, the location and content of m6A in the transcriptome could be accurately identified [88]. In 2023, Liu et al. proposed glyoxal and nitrite-mediated deamination of unmethylated adenosines (GLORI), a high-throughput sequencing-based single-base detection method. The specific mechanism involves employing a reaction system utilizing glyoxal and nitrite as catalysts that transforms conventional adenosine deamination into inosine (A-to-I). During sequencing, isosine is then read as a G, while m6A modification sites are read as an A. Calculating the proportion of the latter allows us to quantify m6A at the single-base level [89].

Furthermore, nanopore sequencing and single-molecule real-time sequencing (SMRT sequencing) are two techniques to identify and quantify m6A modifications by direct sequencing technology [90, 91]. These methods enable high-resolution detection of m6A at the level of individual molecules, making them valuable tools for conducting detailed investigations into the mechanism and function of m6A modifications in RNA biology. Recently, researchers have enhanced the mentioned methodologies by incorporating machine learning algorithms to improve accuracy [92–94].

With technological advances, the detection of m6A modifications has become increasingly precise and efficient. The development of these technologies not only drives advances in epigenomics, but also provides a new perspective for revealing the function of RNA modifications in biological processes. It is worth emphasizing that while high-throughput detection methods offer extensive information on modifications, they are still hindered by challenges related to insufficient sensitivity and specificity. To enhance the reliability of data, researchers can develop more specific antibodies, integrate multiple detection technologies, and implement rigorous quality control measures. Furthermore, advancing data analysis algorithms, including the application of machine learning and artificial intelligence techniques to minimize errors, is a crucial strategy for improving the accuracy of overall analysis. The comprehensive application of these approaches will address current technological limitations and advance m6A research with greater precision.

## m6A biological function

### m6A and mRNA

#### m6A in mRNA splicing

Splicing of precursor mRNA (pre-mRNA) is a pivotal stage in gene expression, and m6A modification has a vital impact on alternative splicing. The m6A locus occurs more prevalent in pre-mRNA than in mature mRNA, with approximately four m6A residue levels of methylation present per pre-mRNA [95]. m6A methylation typically occurs in introns and exons undergoing alternative splicing [69]. YTHDC1 recognizes and binds m6A-modified mRNAs, recruiting proteins belonging to the SR (serine/arginine-rich) protein family, such as SRSF3, to drive exon inclusion. Meanwhile, it inhibits other splicing factors, such as SRSF10, to promote exon exclusion, thereby influencing the outcome of alternative splicing [96–98]. By binding to YTHDC1, METTL3 can indirectly affect alternative mRNA splicing.

METTL16 affects mRNA splicing by regulating intron SAM synthase retention and maintaining low levels of intracellular SAM [99]. The “m6A switch” mechanism describes the role of m6A modification which alters the local RNA structure to change the RNA molecule from a stable structure to a more easily unraveled structure. This mechanism can affect the accessibility of binding sites for HNRNP family proteins (e.g., HNRNPC and HNRNPG), thereby promoting or inhibiting the skipping process of exons [52, 100]. Serial studies in mouse preadipocytes and human 293 T cell lines have shown that FTO impacts splicing factor binding and splice site selection through its demethylation activity. In addition, demethylation of FTO can indirectly regulate alternative splicing of mRNA by altering the interaction between HNRNP family proteins and mRNA [55, 101].

#### m6A in mRNA nuclear export

m6A facilitates the process by which mRNA is transported into the cytoplasm for translation through multiple mechanisms. The process of exporting mRNA from the nucleus is done by the transcription-export complex (TREX), with assistance from the MTC. To promote mRNA nuclear export, the MTC recruits TREX to m6A-modified mRNAs and stimulates their interaction with YTHDC1 [41]. YTHDC1 binds to methylated mRNAs and regulates their subcellular localization. It aids the nuclear export of modified mRNAs by interacting with the nuclear export adaptor protein SRSF3 and the export receptor Nuclear RNA Export Factor 1(NXF1). Knockdown of YTHDC1 results in the retention of m6A-modified mRNA within the nucleus [39]. Additionally, FMRP has the ability to attach to the m6A-modified mRNA, thereby physically bind to the export protein Chromosome Maintenance Region 1 (CRM1), which promoting mRNA nuclear export. However, a conditional knockdown of Mettl14 resulted in blockage of the nuclear export process [102, 103]. There are currently few studies on the nuclear export of mRNAs, and the specific mechanism is still unknown.

#### m6A in mRNA translation

m6A modification is more prevalent in the 5 ʹUTR or 3ʹ UTR of mRNA and affects cyclization of mRNA, thereby promoting translation initiation. The m6A modification in the 5ʹ UTR region has the ability to bind directly to eIF3 and subsequently recognize and bind small ribosomal subunits, initiating cap-independent translation [104]. YTHDF1 enhances translation efficiency and promotes protein production through physical interaction with eIF3. Silencing YTHDF1 results in a down-regulation of protein production of its target mRNA [105]. The interaction of YTHDF3 with YTHDF1 was able to further improve translation efficiency [38]. METTL3-mediated mRNA cyclization is also accomplished by interaction with eIF-associated subunits, and METTL3 enhances the contact of these regions with translation instigation factors to regulate mRNA translation by introducing m6A modifications at the 5 ʹUTR and 3ʹ UTR of mRNAs [104, 106].

The m6A modification located in the 3ʹ UTR region is cap-dependent translation, guided by the interaction between YTHDF1 and YTHDF3 [105]. YTHDF3 stabilizes YTHDF1 binding to mRNA, resulting in enhanced translation [38]. Furthermore, researchers found that YTHDC2 has ATP-dependent RNA helicase activity and enhances target mRNA translational efficiency by encouraging ribosome cycling [107]. Huang et al. proposed that IGF2BPs play a role in regulating stress-related mRNA translation [44]. The m6A modification can also inhibit mRNA translation via related proteins. Interestingly, previous researches have shown that FMRP typically inhibits the translation process of target mRNAs by preventing ribosomal translocation [108, 109]. However, Elias G. Bechara et al. have also proposed that FMRP may positively regulate mRNA translation [110]. As a result, additional investigate is needed to comprehend the precise mechanism of FMRP in regulating of mRNA translation.

#### m6A in mRNA stability

Various kinds of m6A-reading proteins are the main regulators responsible for regulating mRNA stability. Research has demonstrated that IGF2BPs are crucial for the preservation of mRNA stability. By attaching to target mRNAs, IGF2BPs recruit RNA stabilizers and regulators, such as matrix protein 3 (MATR3) and embryonic lethal abnormal vision 1 (ELAVL1), which act together to protect target mRNAs from degradation [44, 111]. YTHDF2-bound m6A mRNA, on the other hand, induces a decay process through two distinct molecular mechanisms [43]. First, the heat-responsive protein (HRSP12) can physically interact with YTHDF2 as an adaptor protein, causing rapid RNA degradation through the endonuclease cleavage pathway. The existence of HRSP12 binding sites in transcripts is essential for this mechanism [112]. Research has demonstrated that it can also stimulate the degradation of circRNAs with m6A modifications [112]. Second, YTHDF2 directly recruits the deadenylase complex CCR4-NOT, triggering deadenylation and initiating the degradation of m6A-modified mRNAs [37, 113, 114]. YTHDF3 can synergize with YTHDF2 to promote RNA decay [38].

Upstream frameshift 1 protein (UPF1) is a key RNA helicase that has been reported to modulate mRNA stability through the nonsense-mediated mRNA degradation (NMD) pathway [115]. YTHDF2 is also involved in this mechanism, further enhancing mRNA degradation by recruiting UPF1. The interaction reflects the synergistic effect of m6A modification with post-transcriptional regulation. Researchers have also reported that YTHDF1 binds m6A-modified mRNA, forming dynamic concentrating compartments in the cell through a liquid-liquid phase separation mechanism to regulate the translation efficiency of mRNA [116–118]. Another reading protein, YTHDC2, has been reported to be able to recruit the exonuclease XRN1 and other RNA degradation complexes, thereby accelerating mRNA degradation [107, 119]. Apart from the aforementioned processes, m6A modification is also crucial for the cytosolic elimination process of non-functional RNAs. RNA degradation complexes degrade m6A by recognizing and labeling nonfunctional RNAs and then binding specific m6A reader proteins [120]. This mechanism guarantees RNA quality control within cells and prevents the accumulation of non-functional RNA that disrupts the normal biological function of cells.

### m6A and rRNA

In eukaryotic cells, the m6A modification of ribosomal RNA (rRNA) is crucial for rRNA processing and translation regulation mechanisms [121, 122]. Several m6A regulators are involved in regulating these processes to ensure precise control of rRNA function and efficient intracellular translation processes. m6A modification on rRNA was first identified in humans and other vertebrates 30 years ago. The METTL5 [27, 123–125] and ZCCHC4 [31, 126] enzymes are both positioned in the nucleolus and specially change the methylation state of 18S rRNA m6A1832 and 28S rRNA m6A4220. Both of them are crucial in regulating specific cellular processes and the biogenesis of ribosomes.

In addition, recent researchers have also proposed that METTL16 participates in translation-related mechanisms during ribosome biogenesis, thereby improving translational efficiency. This process interacts with Subunits of the eIF3 complex, facilitating its binding to 18S rRNA and controlling the translation initiation process [127]. This process is not influenced by its methyltransferase activity. In bacteria, m6A modification similarly influences the translational function of ribosomes and regulates ribosome assembly [126, 128, 129]. Currently, there is a scarcity of research on the correlation between m6A modification and rRNA, so future investigations are anticipated to further reveal the specific mechanism and provide more information for understanding the widespread role of m6A modification in rRNA.

### m6A and lncRNA

At present, the relationship between m6A modification and lncRNAs has garnered significant attention. The m6A modification is vital for regulating the stability and translation processes involved in the biogenesis of lncRNAs, and it affects gene expression through many kinds of complex biological processes. Conversely, lncRNAs can also target m6A-related regulators, which play a crucial role in m6A modification [130–134]. The modification of m6A can alter the structure of local RNAs, thereby inducing RNA-binding protein entry and regulating the function of lncRNAs. For instance, when YTHDC1 recognizes and controls gene silencing caused by long non-coding RNA X-inactivation-specific transcripts (XIST), m6A modification changes the local structure of XIST. This structural change makes it easier for XIST to bind to factors that control silencing and boosts the effect of the X chromosome on silencing [135–137]. Metastasis-associated lung adenocarcinoma transcript 1 (MALAT1) is a lncRNA that is abundantly expressed in the nucleus and up-regulated in neoplastic diseases [138]. The m6A modification regulates MALAT1’s function in splicing by altering its structure, which in turn affects its localization and expression in the nucleus [139]. Correspondingly, MALAT1 affects its own m6A modification level by targeting METTL3 interaction and can also regulate MALAT1 function in regulating gene expression and RNA splicing [137, 139, 140].

In a manner that is dependent on m6A, m6A writers regulate the stability of lncRNA. Several researchers suggest that m6A writers play a necessary role in the progress of gynecology-related malignancies. For instance, METTL3 accelerates cervical cancer progression by promoting m6A modification of the lncRNA FOXD2-AS1 and enhancing the stability of FOXD2-AS1 [141]. In a study based on ovarian cancer, it was discovered that the m6A methylation of lncRNA RHPN1-AS1 enlarged the stability and up-regulation of RHPN1-AS by reducing RNA degradation. Additionally, it promoted the development of epithelial ovarian cancer(EOC) by activating the FAK/PI3K/Akt pathway [142]. Related researches have also discovered the specific mechanisms of m6A writer-related regulators and lncRNAs in Acute myeloid leukemia (AML) [143], hepatocellular carcinoma (HCC) [144, 145], gastric cancer [134], breast cancer [146], and nasopharyngeal carcinoma et al. [147, 148].

In general, m6A methylation of lncRNAs reduces RNA degradation and improves their stability. Some lncRNAs, however, have decreased stability after m6A methylation modification. METTL16 regulates HCC progression by inhibiting the stability of RNA transcripts through the interaction of m6A and RAB11B-AS1 lncRNAs [145]. The newly identified regulator ZCCHC4 can also promote colorectal cancer spread through regulating the m6A modification of lncRNAGHRLOS and inhibiting its stability to downregulate gene expression [149].

m6A readers can recognize and attach m6A modifications, which in turn affect the degradation or stabilization of lncRNAs and regulate tumor development. For example, YTHDC1 recognizes and stabilizes the m6A modification of the lncRNATERRA mediated by METTL3, thereby ensuring the stability of the telomere in cancer cells [150]. IGF2BP1 is an important factor in the initiation and progression of gastric cancer, as it promotes the stability of THAP7-AS1 lncRNA [151]. Recent research has also revealed that m6A erasers are involved in the regulation of the biological function of lncRNAs. For example, FTO-mediated m6A modification of lncRNA LINC00022 improves its stability and stimulates cancer cell proliferation by recognizing YTHDF2 [152]. Another demethylase ALKBH5 increases the stability of the lncRNA KCNK15-AS1, inactivates the PI3K/AKT pathway, and upregulates Phosphatase and tensin homolog (PTEN) expression through a m6A-dependent mechanism, thereby preventing tumor cell proliferation and metastasis [153].

Investigating the regulatory mechanism of m6A modification on lncRNAs can provide valuable insights into RNA functions and the role of RNA modifications in various diseases. In the future, more lncRNAs regulated by m6A modification are expected to be discovered. Further research is necessary to ascertain the precise mechanism of lncRNA biosynthesis and the function of m6A modification in this procedure.

### m6A and miRNA

microRNAs (miRNAs) are a type of small RNA that are endogenously encoded and single-stranded. They are nearly 22–25 nucleotides in length and are produced from primary transcripts (pri-miRNAs) that are transcribed by RNA polymerase II/III [154, 155]. The 3ʹUTR and regions adjacent to the stop codons have abundant binding sites for miRNAs and m6A [68]. Multiple investigations have revealed that the interplay between m6A modification and miRNAs involves regulatory mechanisms at multiple levels, including miRNA biosynthesis, recognition and degradation of target mRNAs, and mutual regulation between miRNAs and m6A-related factors [156].

DiGeorge syndrome chromosome region 8 (DGCR8) plays a key role in primary miRNA processing [157]. According to Ma et al.‘s study, DGCR8 regulates the processing and maturing of pri-miR-126 by recognizing and binding the m6A modification mediated by METTL14 [158]. DGCR8 can also target and process METTL3 methylation-modified pri-miRNAs, as well as promote miRNA maturation [159, 160]. In contrast to the above results, m6A modification is similarly involved in inhibiting pre-miRNA maturation. An osteoporosis-based study found that METTL3 binds to pre-miR-320 and promotes the m6A modification of this molecule. This mechanism prevents osteoporosis by primarily enhancing the differentiation of osteoblasts through the activation of Runt-related transcription factor 2 (RUNX2) [161]. It was discovered in a tumor-based study that METTL3 and METTL14 act on miR-380-3p through a m6A modification-dependent mechanism. This mechanism targets PTEN deleted on chromosome ten to degrade it, resulting in the motivation of the downstream Akt signaling pathway. Consequently, pancreatic cancer progress is promoted [162].

m6A readers regulate miRNA generation and stability and then regulate miRNA target expression by recognizing and binding m6A-modified RNAs. A study by Lyu et al. utilizing ovarian cancer cell demonstrated that WTAP-dependent m6A methylation stimulates the maturation of miR-200 in a DGCR8-dependent manner, ultimately regulating hexokinase 2 (HK2), a vital enzyme in glycolysis, thereby influencing ovarian tumor metabolism and progression. Researchers have also observed that WTAP-mediated m6A modification participates in the promotion of miRNA maturation processes [163]. Another study demonstrated that METTL3/YTHDF2-dependent m6A methylation regulates the expression of the tumor suppressor gene Vasohibin-1 (VASH1) by modulating the interaction between miR-885-5p and lncRNA MEG3[164]. Through bioinformatics analysis, cell function assays, and in vitro models, the study identified a novel mechanism by which miRNAs contribute to ovarian cancer progression [164]. The nuclear reader HNRNPA2B1 is able to specifically recognize and bind certain m6A-modified pri-miRNAs and promote DGCR8-mediated primary miRNA processing, thereby regulating miRNA maturation [51, 165, 166]. Other research indicated that IGF2BPs protein and HNRNPs family protein can function as oncogenes, binding m6A-modified related miRNAs and participating in their maturation process, thereby promoting the development of prostate cancer, liver cancer, and breast cancer [167–169]. Additionally, FTO and ALKBH5 play a significant role in the progression of various kinds of cancer, mainly by controlling the stability of miRNAs and increasing their expression in a m6A-depended way [170–173].

Notably, both of them possess the capacity to function as either a tumor suppressor or a promoter of cancer advancement. Correspondingly, miRNAs can also target associated m6A-modified mRNAs and participate in regulating their stability, thereby regulating m6A modification [174]. For example, miRNAs have the ability to form RNA-induced silencing complexes (RISC), which is a multiprotein complex with endonuclease activity. MiRNAs modulate gene translation by binding to RISC, which then prompts their binding to the 3ʹ-UTR of m6A-modified target mRNAs, thereby mediating the silencing of target mRNAs [175, 176]. MiR-33a targets METTL3-mediated m6A-dependent mRNA, decreases METTL3 expression and result in declining the growth of lung cancer [177]. In conclusion, m6A modification, facilitated by m6A regulators, significantly influences miRNA synthesis via changing miRNA’s biological functions. Further researches are required to elucidate the complex regulatory mechanisms underlying the relationship between these two processes.

### m6A and circRNA

Reverse splicing mechanisms typically form circular RNAs (circRNAs), which are structurally specialised non-coding RNAs that remain unaffected by RNA exonucleases and exhibit greater stability compared to other classes of RNAs [178–180]. CircRNAs’ functionality can be regulated by m6A modification through the regulation of their biogenesis, stability, and translational potential [181, 182]. For example, the METTL3/METTL14 complex adds a m6A modification to pre-mRNA to make it easier to cyclize to generate circRNAs [181, 183]. The reading protein YTHDC1 can promote circRNA maturation by selectively enhancing splicing of circRNAs [184]. YTHDC1 is also involved in circRNA transport [185]. Research has found that YTHDC1 silencing leads to circNSUN2 accumulation in the nucleus, confirming this theory [184]. METTL3-mediated m6A modification facilitates the nuclear export process of circHPS5, thereby mediating hepatocellular carcinoma progression [186].

m6A modification can also drive circRNA translation initiation. This process mainly includes two mechanisms. First, YTHDF1 facilitates the translation of circRNA into functional proteins by enhancing the internal ribosome entry site (IRES)-dependent pathway [187, 188]. Second, the m6A modification can directly start the cap-independent translation of circRNAs by bringing in eukaryotic translation initiation complexes through YTHDF3 [181]. However, FTO-mediated m6A modification inhibits circRNA translation. For instance, m6A demethylation of circDDIT4 represses its expression, thereby promoting the development of prostate cancer [181].

Researchers found that reading protein YTHDF2 is able to bind m6A-modified circRNAs and regulate their stability and degradation. For instance, YTHDF2 controls the m6A modification process of circ0003215 by making it break down faster and lowering the amount of circRNA, which helps colorectal cancer spread [189]. METTL3 is responsible for mediating the m6A modification of circMYO1C to enhance its stability by recognizing IGF2BP2 and playing a role in tumor progression [190]. In contrast, some m6A-modified circRNAs could reduce stability. For example, the demethylase ALKBH5 mediates m6A modification of circNRIP1 to suppress tumorigenesis by inhibiting its stability and downregulating the expression of carcinogenesis-related factors [191].

In the meantime, circRNAs can also target related m6A regulators, directly regulate m6A modification of their target mRNAs [192, 193], or indirectly regulate m6A modification through sponge miRNAs. CircRNAs can specifically bind to miRNAs through multiple binding sites, like how water sticks to a sponge. This stops miRNAs from binding to their target mRNAs, which stops them from doing their biological job [194, 195]. CircPUM1, for example, promotes METTL3 expression via sponge miR-590-5p and has an impact on tumor cell proliferation and glycolytic processes [196]. Examining the interaction between circRNAs and m6A modifications facilitates our comprehension of their biological function and potential applications in disease therapy. Several researches have investigated the influence of m6A modification on circRNAs, yet further research is looked-for to fully comprehend the role of circRNAs in methylation modification.

## Physiological functions of m6A in female reproductive system

### m6A in folliculogenesis and oocyte maturation

The function of m6A modification in folliculogenesis and oocyte maturation is a relatively active area of recent research. During the embryonic stage, primary oocytes within primordial follicles undergo and remain in meiotic prophase I. In preparation for the initial embryonic development, oocytes progressively increase in size and accumulate substantial quantities of mRNA and protein. Oocytes enter metaphase I from meiotic prophase I during follicular maturation, resulting in the formation of secondary oocytes and polar bodies. Secondary oocytes undergo meiotic second division, remain in metaphase II, and complete development prior to ovulation and fertilization [197]. The m6A modification controls the maturation process of oocytes, influences both the stability and translation efficiency of mRNA, regulates the expression of related genes through multiple biological mechanisms involved in precise epigenetic regulation in oocytes.

METTL3 expression is elevated during oogenesis. In a study, siRNA was employed to knockdown Mettl3 in oocytes of the mouse germinalvesicle stage. This caused abnormal spindles, slowed down meiotic progression, reduced the efficacy of mRNA translation, impacted mRNA degradation, and halted oocyte development [198]. Supporting this conclusion, an additional investigation discovered that METTL3 mutations impeded the initial stages of oocyte development and resulted in a substantially lower percentage of fully developed follicles than controls. Intersectin 2 (ITSN2) is a multifunctional protein that is involved in the process of meiotic resumption in oocytes [14]. METTL3 identifies and attaches to ITSN2 and carries out m6A alteration, which can increase its durability and facilitate oocyte development. Specific Mettl3 knockout in mouse oocytes causes meiotic maturation failure [13]. Also, the development of oocytes is halted and adult females become less fertile when mettl3 mutations are detected in zebrafish. This could be due to the global decrease in m6A levels and the blockage of critical genes that are necessary for the production of hormones and gonadotropins [199]. A study conducted on oocytes from pigs revealed that the methylation of m6A by METTL3 and WTAP has the potential to influence the maturation of oocytes and the growth of embryos [200].

The reading proteins YTHDC1 and YTHDC2 are required for oocyte maturation. Seth D. Kasowitz et al. engaged in a mouse study in which the inactivation of YTHDC1 led to mRNA defects in alternative splicing in mouse oocytes. The production of extensive variable polyadenylation altered the 3ʹUTR length, thereby hindering oogenesis [201]. YTHDC2-deficient female mice have small ovaries and lack fertility [202]. YTHDF2 regulates the degradation of maternal mRNA during MII [203]. Knocking down YTHDF2 modifies the amount of transcription of genes that affect oocyte development, thereby affecting the quality of oocytes. In mice, the absence of YTHDF2 results in the failure of oocyte maturation and female-specific infertility. KIAA1429 controls the alternative splicing of genes involved in oocyte maturation. Conditional knockout mice display Kiaa1429 in their oocytes, resulting in female mice becoming infertile [204]. The IGF2BPs family has a role in oocyte maturation by engaging in the m6A methylation process of RNA and enhancing mRNA stability [13]. Additionally, a transcriptome-wide investigation of chicken follicles by Yu et al. indicates that the follicle selection process may be influenced by m6A modification [205].

### m6A in embryonic development

Embryonic development commences with fertilization, during which the zygote exhibits a transcriptionally quiescent state. Following this, the maternal-to-zygote transition (MZT) occurs, which involves the activation of the zygote genome (ZGA) and the elimination of maternal storage (RNA and DNA) [206, 207]. The importance of RNA m6A modification in the development of mammalian embryos has been increasingly recognized in a variety of studies [208]. It has been reported that the degradation of maternal mRNA is impeded by the loss of Mettl3 in mouse oocytes, which in turn affects embryonic developmental progression by impeding MZT and zygotic genome activation [198, 209]. m6A methylation can also influence embryonic development by modulating the stability of mammalian embryonic stem cells (mESC). For example, the absence of Mettl3 results in the loss of the self-renewal and differentiation capabilities of rodent embryonic stem cells [210, 211]. Relevant demonstrated that the Mettl3 gene deletion in mammals results in embryonic mortality [212, 213].

Mettl14 deficiency substantially delays early embryonic development and results in embryonic death, primarily by obstructing the process of ectodermal cell differentiation to maturation [214]. Furthermore, a study conducted by Liu et al., which involved the construction of an embryonic mouse model lacking Mettl16, discovered that the absence of Mettl16 resulted in a reduction in the mRNA levels of its methylation target Methionine adenosyltransferase 2 A (MAT2A) and the obstruction of embryonic development. This finding underscores the essential role of Mettl16 during the initial phases of embryonic development [29]. Researchers have also shown that the recently discovered writing protein Mettl5 affects embryogenesis by participating in the m6A modification of rRNA and regulating pluripotency in mouse embryonic stem cells (mESC) [123].

As mentioned above, YTHDC1 not only regulates oogenesis but also participates in the self-renewal and differentiation processes of mouse embryonic stem cells, which affects embryonic development [201]. Studies have shown that YTHDC2-knockout mice survive, but their adult counterparts are infertile [202]. Suppression of the IGF2BP2 protein results in the halting of mouse embryos at the 2-cell stage and hinders the advancement of early embryonic development [215]. A study discovered that the inhibition of YTHDF2 in goat embryonic cells resulted in the prevention of maternal mRNA degradation and impaired zygotic genome activation. It was hypothesized that YTHDF2 influences mRNA clearance by controlling the expression of adenosine deaminase and mRNA decapping enzyme [216]. Studies using mouse and zebrafish have demonstrated that the deletion of the maternal YTHDF2 gene significantly hinders the development of embryos [217, 218].

Furthermore, the demethylase ALKBH5 primarily controls the development of the male testes [219]. FTO in mESC is responsible for the demethylation modification of long interspersed element 1 (LINE1) RNA, which has an impact on embryonic development, growth, and differentiation of embryonic stem cells [220]. To understand the complex mechanisms of gynecology-related diseases, it is essential to conduct a more thorough investigation of the molecular mechanisms of the connection between the m6A modification and embryonic development.

### m6A in placental development

The placenta acts as a bridge between the mother and the baby, performing tasks like immunological modulation, nutrition exchange, and stress response. It also plays an essential role in preserving pregnancy and the growth of the fetus [221, 222]. Placental cells, particularly trophoblast cells, are highly proliferative and invasive. The modulation of the maternal immune system and gene expression is facilitated by their involvement in numerous mechanisms, which are designed to ensure the seamless implantation and development of the embryo in utero [223, 224]. Furthermore, the placenta, functioning as a physiological neoplasm, is connected to cancer through multiple processes [225]. The majority of m6A modification sites are situated in introns of human placental tissue and are enriched near stop codons [208, 226, 227]. The methylation modification of m6A significantly influences the biological function of placental cells.

By increasing the stability of placental villous trophoblast cells, the m6A methylation of MYLK mRNA by methyllase-METTL3 can affect their invasiveness. Additionally, it is implicated in the regulation of preeclampsia [228]. YTHDC1 is involved in the m6A methylation modification of circMPP1, which leads to a decrease in its expression and the inhibition of placental function. This process is regulated by both the NF-κB and MAPK3 signaling pathways [229]. Through an analysis of the m6A modification levels at the 5ʹ-UTR of placental mRNA in infants with varying birth weights, one study suggested that m6A modification may regulate placental function and influence fetal growth by participating in translational regulation [208]. Through the development of a BaP-exposed mouse model, Wang et al. discovered that lnc-HZ14 induces early abortion and promotes placental trophoblast pyroptosis by participating in and increasing the METTL3-mediated m6A modification of pyridine domain-containing 3 inflammasome (NLRP3) mRNA in the NOD-like receptor (NLR) family [230].

The demethylase ALKBH5 is an important regulator of placental development. Knocking down ALKBH5 can promote trophoblast invasiveness and result in recurrent miscarriage, as it can modify human trophoblast invasiveness by regulating CYR61 mRNA stability [231]. Another study also verified the involvement of ALKBH5 in the progress of preeclampsia by modulating the m6A methylation modification of PPARG mRNA [232]. Placental dysplasia-related proteins and targets are important for the early diagnosis and management of gynecological diseases. Nevertheless, there are limited relevant studies, and their specific mechanisms require further investigation.

### m6A in maternal-fetal microenvironment

**Fig. 2:**
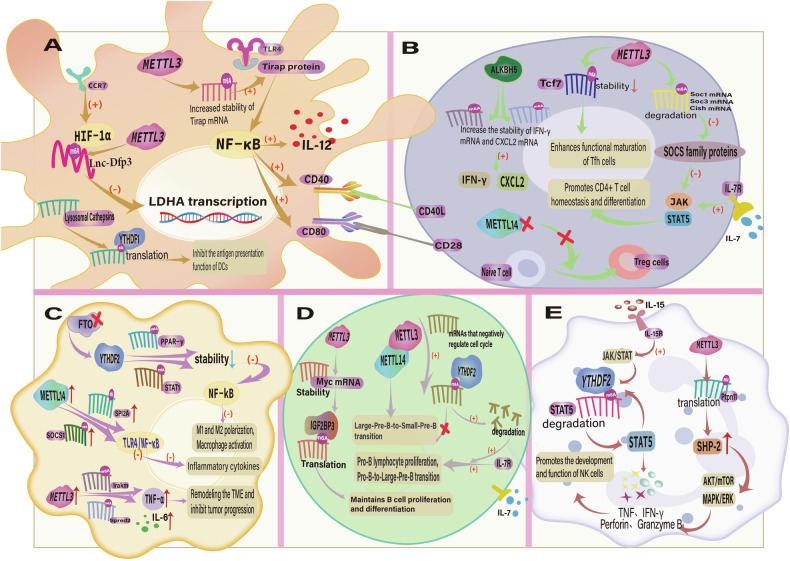
m6A modification regulates the function of immune cells in the maternal-fetal microenvironment through multiple mechanisms. **A** In DCs, m6A methylation regulates the translation of proteases in CD40, CD80, and lysosomes, thereby promoting DC maturation and inhibiting antigen presentation. METTL3 influences the energy metabolism of DC by regulating the expression of HIF-1 and the glycolytic gene LDHA. **B** by modulating SOCS family mRNA and the IL-7/JAK/STAT5 pathway, METTL3 maintains CD4 + T cell homeostasis. Additionally, it is essential for the development of Tfh cells. METTL14 deficiency results in the blocking of Treg cell differentiation. **C** In macrophages, METTL3 regulates the tumor microenvironment (TME), disrupts TLR4 and ERK signaling pathways, affects Irakm and Spred2 expression, and inhibits tumor progression. **D** m6A modification plays an important role in various developmental stages of B cells. **E** NK cell function is regulated by the degradation of STAT5 mRNA and the expression of SHP-2, which are regulated by YTHDF2 and METTL3, respectively.

The maternal-fetal microenvironment is a complex biological interface between the mother and fetus. Its main role is to support the embryo’s growth and development while protecting it from harmful substances and pathogens. Furthermore, the occurrence of related diseases is closely correlated with alterations in the maternal-fetal interface microenvironment [233]. Germ stem cells are implicated in mechanisms related to immune tolerance and fetal development in the maternal-fetal microenvironment. The study of cancer stem cells is provided with a distinctive perspective by the similarity between the maternal-fetal microenvironment and the tumor microenvironment in certain regulatory pathways and mechanisms. The normal development of embryos is ensured by the interaction between multiple important immune cells, which is crucial in the mother and fetus’s mutual equilibrium of immunological tolerance and protection (Fig. 2). Cross-sectional research in these regions offer substantial support for comprehending the operation of the female reproductive system.

#### m6A and germline stem cells

Female germline stem cells (FGSCs) are a kind of stem cell that are found in the female reproductive system of mammals. They possess the ability to self-renew and develop into fully mature oocytes [234, 235]. The relationship between FGSC and m6A modification has garnered a growing amount of attention in recent years. Zhao et al. performed a comparative investigation of the m6A methylation levels of mRNAs in female mouse germline stem cells and thioguanine and ubarine-resistant (STO) cells using MeRIP-seq. They discovered that the former had significantly higher overall m6A levels than the latter. Subsequent investigations indicated that the proliferation process of FGSC was influenced by the reading protein YTHDF1, and the self-renewal capacity of FGSC was substantially reduced by a specific knockdown of YTHDF1 [236].

In addition, correlational research has suggested that the m6A modification of non-coding RNAs is associated with FGSC. For instance, the nuclear export progression of circGFRα1 is influenced by the m6A modification mediated by methylase METTL14, which in turn regulates GFRα1 expression and influences self-renewal in FGSC [237]. Currently, the specific relationship between m6A modifications and FGSCs is being investigated; however, it is conceivable that m6A modifications may be crucial in the preservation of the self-renewal and differentiation processes of FGSCs. We can anticipate a more comprehensive comprehension of this field as science and technology continue to advance and research continues to deepen.

#### m6A and cancer stem cells

Cancer stem cells (CSC) are present in various of tumor types and possess stem cell characteristics. They are vital in tumor progression, recurrence, drug resistance, and metastasis of tumors [238–240]. The invasiveness of the placenta is somewhat similar to the mechanisms associated with CSCs, which also suggests a potential role for the maternal-fetal microenvironment in the development of tumors. Numerous studies have documented a correlation of m6A and tumor resistance, tumor epithelial-mesenchymal transition (EMT), and self-renewal and differentiation of cancer stem cells [241–243]. Currently, there has been a growing interest in the close correlation between CSC and m6A modification. Numerous malignancies including breast cancer [244], lung cancer [245], cutaneous squamous cell carcinoma [246], acute leukemia [247], and colon cancer [111], have been disclosed to play critical functions in m6A modification. Gradually, studies that investigate the relationship between gynecological malignancies and CSC are also being conducted.

A study of cisplatin-resistant ovarian cancer cell lines discovered that YTHDF1 was capable of resisting ovarian cancer cells by improving their CSC-like characteristics and promoting tumor progression by binding to m6A-modified three-part motif protein 29 (TRIM 29) and enhancing its translational efficiency [248]. In cervical cancer, overexpression of TRIM 29 also has a carcinogenic effect, but more researches are required to determine the complete mechanism [249]. Based on an investigation conducted by Chen et al., hypoxia induces the demethylase ALKBH5, which in turn regulates the m6A methylation of the transcription factor SOX2 mRNA and promotes the properties of endometrial cancer stem cells by reducing its methylation level. Hypoxia-inducible factor downregulated the level of ALKBH5 protein, which in turn inhibited the tumorigenicity of CSCs, thereby confirming the aforementioned conclusion [250]. A comprehensive understanding of the characteristics and mechanisms related to CSC could enhance patient survival, reduce tumor recurrence and metastasis, and contribute to a more effective clinical battle against cancer.

m6A modification not only influences the biological behavior of tumor cells but also regulates key processes within the tumor microenvironment (TME), including the hypoxic response, metabolic reprogramming, cell cycle regulation, and immune cell function. In the immune microenvironment, tumor cells depend on glycolysis to sustain their energy supply [251]. A study by Li et al. employed Mettl3-knockdown cervical cancer cell lines (HeLa and SiHa) alongside the human normal cervical epithelial cell line ECT1/E6E7 for in vivo and in vitro assays. The results indicated that YTHDF1 and IGF2BP3-dependent m6A methylation positively regulates glycolysis in cancer cells, thereby promoting tumor growth and progress by enhancing the stability and translation efficiency of PDK4 mRNA [252]. Additionally, another study unveiled that METTL3 regulates CDC25B translation through m6A modification-dependent YTHDF1. This regulation was demonstrated through a comprehensive approach, including in vitro cervical cancer cell cycle synchronization, m6A-seq analysis, in vitro cell proliferation assays, and in vivo nude mouse tumor formation experiments, ultimately promoting cell cycle progression [253]. These findings highlight that m6A modification-related mechanisms hold promise as potential therapeutic targets for cancer in the future.

#### m6A and DC cells

Dendritic cells (DCs) are antigen-presenting cells (APCs) which perform vital immunoregulatory functions in the maternal-fetal microenvironment and are instrumental in the emergence and development of tumors [254, 255]. Krey et al. developed a transgenic mouse model of DC depletion and discovered that DC deficiency prevented the embryo from implanting and resulted in aberrant vascular development in the uterine decidua. This affected the process of placenta development and the subsequent pregnancy [256]. In the past few years, m6A modification and DCs’ role in the immune microenvironment have gained increasing attention. As early as 2005, researchers proposed that the innate immune response process could be influenced by the m6A modification of RNA, which could reduce DC-mediated Toll-like receptor (TLR) expression [257]. Wang et al. discovered that the methylase Mettl3-mediated m6A modification may boost the expression of associated immune costimulators, increase their translational efficiency, activate DCs, and encourage the initiation of T cells. The TLR4/NF-κB signaling pathway is activated during this process, which results in the production of cytokines. Mettl3 knockdown then impedes this process, which impacts the normal function and maturation processes of DCs [258].

The YTHDF1 reading protein is responsible for the regulation of lysosomal proteases and the facilitation of tumor immune escape. YTHDF1 reduction enhances the capacity of DCs to present tumor antigen by decreasing its antigen consumption [259]. The putative function of YTHDF2 as a tumor immunosuppressive factor is reflected in the mechanism by which it recognizes and binds the m6A modification site of lnc-Dpf3 in DCs, thereby inhibiting DC migration [260]. To summarize, targeting m6A in DCs has great promise for cancer immunotherapy. Furthermore, the association between DC and m6A modification in obstetrics and gynecology is poorly researched, which underscores the need for additional clarification.

#### m6A and NK cells

Uterine natural killer cells (uNK cells) are a distinct type of immune cell that are present on the endometrium. They are collectively responsible for the preservation of immune homeostasis at the maternal-fetal interface. Former research has shown that the development of the placenta is contingent upon the presence of uNK cells. [261], the growth of the fetus [262], the modulation of inflammation and the immune system [263], and the mechanisms related to tumors [264]. Decidual NK (dNK) cells represent a specific subtype of uNK cell that are predominant in the early maternal-fetal interface of humans [265, 266]. dNK cells also play a critical role in keeping maternal-fetal microenvironment homeostasis. The significance of m6A modification in modulating the activity of NK cells biological function has steadily become an increasingly hot topic, and the interaction between the two in gynecological diseases is also a developing research area.

It has been reported that the process of NK cell maturation is facilitated by the m6A modification mediated by the reading protein YTHDF2. The antitumor properties of NK cells were subsequently inhibited by the specific knockdown of YTHDF2 [264, 267]. The function of METTL3-related m6A modification in regulating NK cell killing activity has also been confirmed [268]. Furthermore, a study conducted a bioinformatics analysis of the m6A target gene cell division cycle 42 effector protein (CDC42EP3) in ovarian cancer and discovered that its down-regulation was associated with NK cell infiltration and the promotion of ovarian cancer progression. This identified a potential association between NK cells and m6A modification in ovarian cancer [269].

In contrast, demethylase FTO has an adverse impact on NK cells, and the specific reduction of FTO results in increased NK cell activation and a decreased risk of melanoma progression [270]. The diagnosis and treatment of gynecological diseases is also likely to be influenced by the specific mechanism of m6A modification in NK cells. Therefore, it is critical to have a comprehensive understanding about this mechanism.

#### m6A and macrophages

Decidual macrophages are responsible for the primary maintenance of immune tolerance at the maternal-fetal interface during pregnancy by secreting anti-inflammatory cytokines, including IL-10 and CCL2, which counteract the maternal immune system’s attack on the fetus [271]. The critical function of macrophages in immune regulation [272, 273], trophoblast development [274], fetal growth [275], and tumorigenesis [276] has been showed in previous studies. The m6A modification has the capacity to regulate the polarization status (M1 and M2 macrophages) and biological function of macrophages by modulating gene expression mechanisms [277].

Several investigations have confirmed that the methylase METTL3-mediated m6A modification may increase the expression of the transcription factor STAT1, thereby positively influencing M1 macrophage polarization while negatively affecting M2 macrophage polarization [278, 279]. YTHDF2 is also involved in the regulation of m6A modification of STAT1 mRNA and the promotion of its degradation, which in turn promotes the polarization of M1 macrophages. This process is accomplished by interacting with the RNA-binding motif 4 (RBM4) [280]. Nevertheless, a study discovered that lactate could stimulate the production of Mettl3-mediated m6A modification, which then positively regulated the polarization of M2 macrophages and resulted in the invasion of endometriosis [281].

In addition, the interaction between m6A modification and tumor-associated macrophages (TAM) is becoming increasingly significant in the modulation of tumor immunity and drug resistance. For instance, ALKBH5 has the potential to influence the homeostasis of the tumor microenvironment by modulating macrophage polarization and function. Jiang et al. conducted an in vitro simulation of the tumor microenvironment by cultivating ovarian cancer cells and M2 macrophages. They discovered that both demethylase ALKBH5 and TLR4 expression were increased, confirming that ALKBH5-mediated m6A demethylation modification activates the NF-κB pathway, which promotes the invasiveness of ovarian cancer [282].

Furthermore, the significance of the interaction between TAM and the ovarian cancer microenvironment has been elucidated in related studies, which are intended to investigate resistance-associated targets and recurrence markers [283]. In cervical cancer, a study suggested that the glycolytic process is targeted, programmed cell death protein 1 (PD-1) expression is facilitated, and macrophage phagocytic function is inhibited by high expression of the m6A regulator METTL14, which accelerates cancer progression [284].

Therefore, the disclosure of the comprehensive effect of m6A modification on macrophage function could enhance comprehension of the role of macrophages in tumor microenvironment and gynecology-related immune responses, thereby facilitating the development of immunotherapeutic strategies that target m6A modification.

#### m6A and T cells

The body’s immune balance is maintained by the synergistic action of various subsets of T cells, which are critical factors of the adaptive immune system, in recognizing and responding to pathogens and tumor cells [285]. m6A modification has demonstrated a significant role in the maternal-fetal interface by modulating T cell development, differentiation, and function [286].

Li et al. identified that the methylase METTL3-mediated m6A methylation was capable of targeting the IL-7/STAT5/SOCS pathway and influencing mRNA degradation processes, thereby modulating the proliferation and differentiation of CD4 + T cells [287]. Also, this post-transcriptional regulation, which is activated by METTL3, positively influences the differentiation of T follicular helper (Tfh) cells [288]. Regulation T (Treg) cells are induced at the maternal-fetal interface to safeguard the fetus from immune rejection and are also closely linked to recurrent miscarriage and infertility [289, 290]. A study conducted by Tong et al. discovered that Mettl3 KO mice exhibited infertility as a result of autoimmune defects, and they specifically lost the suppressive function of Tregs in comparison to their WT littermates. This result also serves as confirmation of the essential regulatory function that METTL3 serves in the preservation of the immunosuppressive function of Treg cells [291].

In ovarian cancer, the C3a anaphylatoxin chemotactic receptor (C3AR1) is associated with the activation of T cells and the penetration of immune cells by mediating m6A modification in OC cells, which results in the development of ovarian cancer [292]. An in-depth examination of the molecular mechanisms involved in T cell modification with m6A could aid in the comprehension of the intricate regulatory network of the immune system and potentially offer new strategies and targets for the medical management of maternal-fetal-related diseases.

#### m6A and B cells

B cells are mainly involved in humoral immunity, which involves the production of specific antibodies to neutralize pathogens and functions by recognizing antigens [293]. Currently, there is a limited number studies examining the correlation between B cells and the maternal-fetal immune microenvironment. According to reports, trophoblast glycoprotein (TPG) is capable of binding B cell receptors and inhibiting their activation, which is crucial for the promotion of maternal-embryonic immune tolerance and the maintenance of normal fetal development [294]. Additionally, a study found that decidual B cells can prevent spontaneous preterm birth by interfering with the PIBF1 pathway, which is regulated by interleukin 33 (IL-33) [295].

Related research has indicated that m6A modification may be a critical factor in the early progress of B cells. For instance, METTL14-mediated m6A methylation is involved in B cell maturation, whereas YTHDF2 reduces interleukin-7 (IL-7)-induced pro-B cell proliferation by binding to m6A modification sites and degrading specific mRNA transcripts, thereby influencing early B cell transformation processes [296]. This process is not regulated by YTHDF1 [296].

Furthermore, the development and prognosis of acute lymphoblastic leukemia [297], human glioma cells [298], and other conditions may be influenced by aberrant methylation modification of m6A in B cells. A study conducted a biological information analysis to screen m6A-related lncRNA prognostic genes in patients with endometrial cancer of the corpus uteri (UCEC). The results indicated that B-cell immune infiltration scores were elevated in the high-risk group than in the low-risk group of the disease, suggesting a possible role for B cells in the progression and prognosis of UCEC [299]. Currently, the investigation of B cells in epigenetic modifications is in its infancy, necessitating the subsequent undertaking of more comprehensive studies on gynecology-related diseases that are founded on the interaction between m6A and B cells.

#### m6A and female reproductive hormones

Female reproductive hormones are indispensable for the modulation of reproductive system function, the maintenance of reproductive health, and the regulation of the female reproductive cycle [300]. The biological roles of the female reproductive system and the development of related diseases have progressively received attention to the regulation of reproductive hormones by m6A modification. However, there are limited studies in this field. Previous sections have indicated that the suppression of METTL3 has the capacity to modulate the expression of genes that are essential for the synthesis of sex hormones, thereby contributing to oocyte development [199]. Gonadotropin-releasing hormone (GnRH) is a decapeptide hormone secreted by the hypothalamus that affects the progression and function of the female gonad by regulating gonadotropin (LH and FSH) secretion from the anterior pituitary gland.

Pulsatile release of GnRH is indispensable for the preservation of typical reproductive function [301, 302]. A study discovered that the demethylase FTO enhances the stability of Foxp2 mRNA and mediates m6A demethylation, thereby promoting gonadotropin synthesis and secretion by up-regulating the cAMP/PKA signaling pathway [303]. According to a study accompanied by Wan et al., the methylation modification of m6A by METTL3 can be instrumental in the regulation of the mRNA stability of the estrogen response genes Elf3 and Celsr2 and the maintenance of the expression level of the progesterone receptor PR. This modification is vital for the regulation of intrauterine estrogen and progesterone signaling. Knockdown of METTL3 leads to infertility [304]. Another study also confirmed that METTL3-mediated m6A modulates the process of mbryo implantation by affecting progesterone signaling through the regulation of the progesterone receptor gene (Pgr) mRNA translation process [305].

Additionally, Mettl14 is involved in the modulation of embryo implantation through a m6A modification-dependent pathway, a process that involves immune-related mechanisms and estrogen receptor α (ERα) signal transduction pathways [306]. Xue et al. also confirmed that FTO-mediated m6A demethylation is involved in the development of polycystic ovary syndrome by modulating the androgen receptor/protein kinase B (AR/AKT) pathway, which is responsible for affecting androgen levels in granulosa cells and follicular fluid [307]. A thorough investigation of the precise function of m6A modification in the synthesis and secretion of female reproductive hormones can facilitate the exploration of the pathogenesis of reproductive system diseases and the development of innovative strategies for their diagnosis and treatment.

## m6A in obstetrics and gynecology-related diseases

**Fig. 3:**
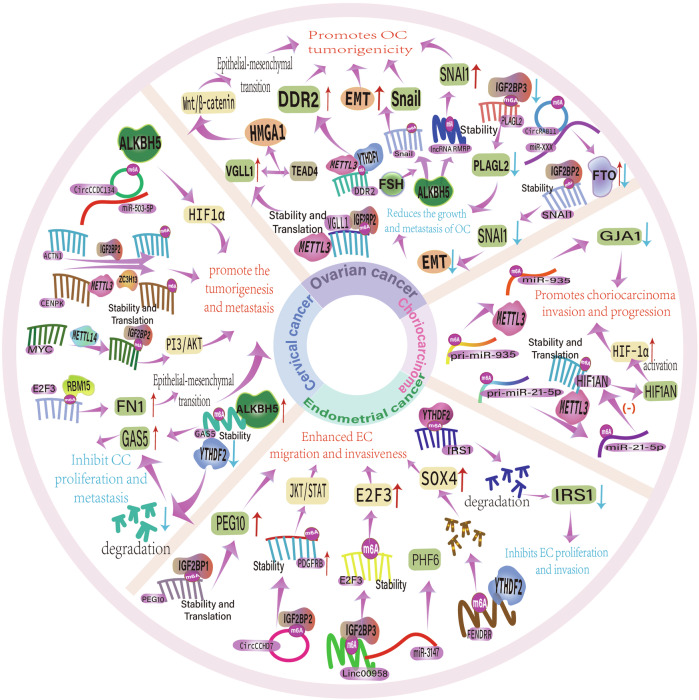
Effect of m6A modification on gynecological malignancies. In cervical cancer, m6A modification-associated transcripts consist of mRNA (ACIN1, CENPK, E2F3, GAS5, MYC), circCCDC134, and miR-503-5P. In ovarian cancer, m6A modification-associated transcripts consist of mRNAs (VGLL1, DDR2, Snail, PLAGL2), CircRAB11 and its target-related miRNAs, and lncRNA RMRPs. In endometrial cancer, m6A modification-associated transcripts consist of mRNA (PEG10, PDGFRB, E2F3, FENDRR, IRS1), Circcchd7, miR-3147, and Linc00958. In choriocarcinoma, transcripts associated with m6A modification include HIF1AN, miR-935, and miR-21-5p.

**Fig. 4:**
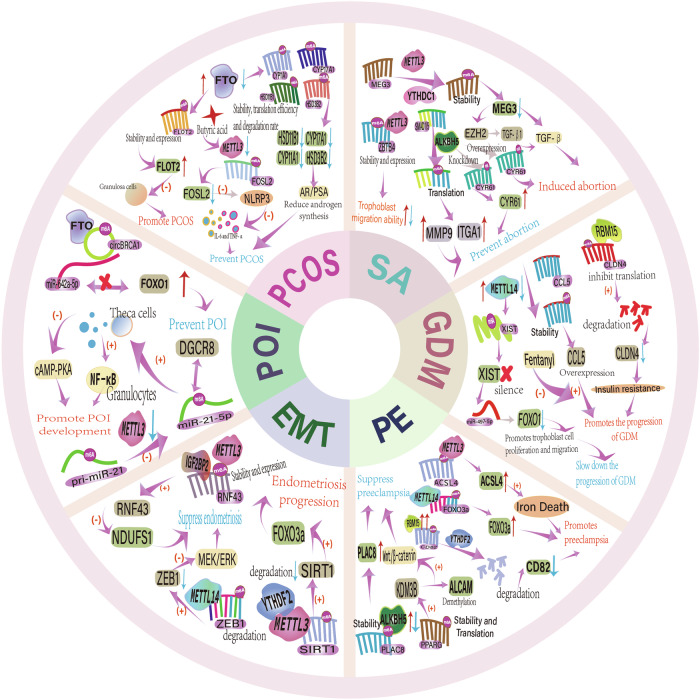
Effects of m6A modification on benign gynecological and obstetric diseases. Numerous gynecological disorders, such as endometriosis (EMT), polycystic ovary syndrome (PCOS), premature ovarian failure (POI), spontaneous abortion (SA), gestational diabetes mellitus (GDM), and preeclampsia (PE), have been found to be influenced by m 6 A. In the aforementioned disorders, we describe modifications in the m 6 A methylation-associated enzymes METTL3, METTL14, RBM15, YTHDC1, YTHDF2, IGF2BP2, FTO, and ALKBH5.

The m6A modification is a critical factor in the development of both benign and malignant female reproductive system diseases. By modulating the expression of genes that are linked to malignancies, m6A modification influences the growth and metastasis of cervical cancer (CC), endometrial cancer (EC), choriocarcinoma (CCA), and ovarian cancer (OC) (Fig. 3, Table 1). In the context of benign disorders, m6A modification is implicated in PCOS, primary Ovarian Insufficiency (POI), spontaneous abortion (SA), gestational diabetes (GDM), and preeclampsia (PE) through a variety of underlying mechanisms (Fig. 4). Consequently, comprehensive investigations of m6A modification mechanisms not only enhance the understanding of the pathogenesis of these diseases but also offer robust support for clinical decision-making.

**Table 1:** The roles of m6A in gynecological cancer.

Disease	m6A regulator	Expression	Target	Mechanism	Ref
**Cervical cancer**	METTL3	Up	IRF5, PPP6C	Increase PPP6C expression level and promote the progression of CC.	[310]
	METTL3	Up	ACIN1	Enhance the stability of ACIN1 mRNA and accelerate the proliferation and migration of CC.	[452]
	METTL3	Up	HK2	Targeting the 3ʹ UTR of HK2 mRNA, enhances HK2 stability and the Warburg effect.	[312]
	IGF2BP2	Up	MYC	Promote aerobic glycolysis, proliferation and metastasis of CC cells.	[314]
	ALKBH5	Down	SIRT3, ACC1	Promote SIRT3 instability, down-regulate ACC1 level, and inhibit tumorigenesis and lipid metabolism.	[315]
	METTL14	Up	TRIM11, PHLPP1	Enhance TRIM11 mRNA stability, PHLPP1 is reduced by ubiquitination, and activated AKT signaling pathway.	[316]
	YTHDF2	Up	AXIN1	Regulates tumor EMT and cisplatin chemotherapy sensitivity.	[320]
	FTO	Up	BMP4	BMP4 is upregulated in an m6A-dependent manner, activating the Hippo/YAP1/TAZ signaling pathway and promoting tumor invasion.	[321]
	ALKBH5	Up	circCCDC134	Regulates MYB expression, activates HIF1A transcription, and exerts oncogenic effects.	[323]
**Ovarian cancer**	METTL3	Up	AXL	Upregulated AXL to boost EMT.	[332]
	METTL3	Up	VGLL1, HMGA1	VGLL1 interacts with TEAD4 to activate HMGA1, which in turn activates the Wnt/β-catenin signaling pathway and promotes EMT.	[333]
	METTL14	Down	TROAP	Enhance TROAP expression levels and accelerate OC aggressiveness.	[334]
	WTAP	Up	MAPK, AKT	Regulates MAPK/AKT signaling pathways, promotes cell proliferation and migration.	[429]
	KIAA1429	Up	ENO1	Stabilize ENO1 mRNA in an m6A-dependent manner to promote tumor progression and glycolysis.	[337]
	HNRNPA2B1	Up	miR-30c-5p	miR-30c-5p downregulates the level of HNRNPA2B1, thereby inhibiting OC cell proliferation.	[338]
	YTHDF1	Up	EIF3C	Enhances EIF3C translation through m6A modification and promotes protein synthesis, leading to OC proliferation and metastasis.	[339]
	YTHDC1	Down	PIK3R1, GANAB	Affect N-glycosylation biosynthesis, inhibit the development of OC.	[341]
	FTO	Down	SNAI1	Inhibits tumor cell EMT and slows down tumor growth and metastasis.	[345]
**Endometrial cancer**	METTL14/ METTL3	Down	PHLPP2, mTORC2	Activates the AKT signaling pathway, promotes tumor proliferation.	[350]
	YTHDF2	-	YBX2, HSPA6	YTHDF2 interacts with YBX2 in an m6A-dependent manner to co-regulate HSPA6 mRNA stability.	[353]
	YTHDF2	Up	IRS1	Downregulated IRS1 expression, inhibit IRS1/AKT signaling pathway and EC proliferation.	[354]
	IGF2BP1	Up	PEG10, p16, p18	Enhance PEG10 mRNA stability, inhibit the expression of p16 and p18, promote EC proliferation and regulate tumor cell cycle and cancer progression.	[355]
	WTAP/ IGF2BP3	Down	EGR1, PTEN	Promotes the formation of EC stem cell properties and the development of tumors.	[359]
	IGF2BP3	Up	E2F3, LINC00958	Upregulated E2F3 expression and promoted the progression of EC.	[361]
	ALKBH5	Up	IGF1R	Promotes the translation of IGF1R protein and EC proliferation.	[363]
**Choriocarcinoma**	METTL3	Up	GJA1, miR-935	Promote the maturation of miR-935, inhibit the expression of GJA1, and promote the proliferation and migration of CCA.	[367]
	METTL3	Up	HIF1AN, HIF1A, VEGF, miR-21-5p	Activate HIF1A/VEGF pathway to promote EMT of CCA.	[368]

### m6A in cervical cancer

CC is a public health issue that poses an imperative threat to the reproductive health of women and is the fourth most widespread form of cancer in women worldwide. Cervical malignancy is primarily caused by an insistent infection with high-risk HPV. However, the prevention of cervical cancer has been demonstrated to be effective through regular screening and prophylactic vaccination [308, 309].

m6A-related regulators are crucial to the progression and development of CC. METTL3 regulates the stability and translation of the catalytic subunit of protein phosphatase 6 (PPP6C) through the m6A modification of mRNA, thereby influencing the downstream transcription factor interferon regulatory factor 5 (IRF5) activity and collaboratively regulating the malignant biological behavior of cervical cancer [310]. METTL3 and IGF2BP3 also regulate the expression level of apoptotic chromatin condensation inducer 1 (ACIN1). The specific mechanism by which ACIN1 induces CC invasion may be accomplished through the interaction of these two m6A regulators, thereby up-regulating the expression level of ACIN1 [311].

The role of regulators related to m6A has garnered increasing attention in tumor metabolism, including glycolysis and lipid metabolism. A study by Wang et al. discovered that METTL3 targets and increases the m6A modification of hexokinase 2 mRNA, while recruiting YTHDF1 to recognize the m6A modification sites on the transcript, enhancing mRNA stability and thus promoting the occurrence of CC and glycolysis [312]. METTL14 and IGF2BP3 have the potential to maintain the stability of stearoyl-CoA desaturase (SCD) mRNA through the m6A system, which can influence lipid metabolism processes and provide new therapeutic strategies for CC [313].

Additionally, numerous investigations have successively documented the precise mechanism by which IGF2BP2, ALKBH5, and other compounds regulate the metabolism of cervical malignancies via the m6A mechanism [252, 314, 315]. Zhang et al. discovered that METTL14 and IGF2BP1 mediate the m6A modification of TRIM11 (an E3 ubiquitin ligase) mRNA, thereby increasing its stability and expression levels. The upregulation of TRIM11 promotes the degradation of the PH domain and leucine-rich repeat protein phosphatase 1 (PHLPP1), which in turn contributes to the progression of CC [316]. In cervical cancer, the activation of the PI3K/Akt pathway is frequently associated with tumor aggressiveness and poor prognosis [317]. Li et al. demonstrated that METTL14-mediated m6A modification regulates the stability of FTH1 mRNA, influencing the PI3K/Akt signaling pathway and thereby inhibiting the progression of cervical cancer [318].

YTHDF1, a reading protein, identifies and binds the m6A modification on RANBP2 mRNA, thereby improving its translational efficiency and facilitating the development of CC [19]. According to a study investigated by Tao et al., YTHDF2 initially recognizes the m6A modification of Nuclear Receptor Subfamily 4 Group A Member 1 (NR4A1) mRNA, that is mediated by METTL3. Subsequently, it collaborates with the RNA helicase DDX6 to further enhance RNA degradation and decrease NR4A1 expression, thereby propelling CC progression [319]. Another study also demonstrated that YTHDF2 plays a role in regulating the expression of the target gene AXIN1, which indirectly influences the activity of the Wnt/β-catenin signaling pathway. This, in turn, modulates the EMT progression in cervical tumors and affects the sensitivity to chemotherapeutic agents [320]. Huang etal.‘s research in cervical carcinogenesis proved that FTO accelerates tumor progression by eliminating m6A methylation from Bone Morphogenetic Protein 4 (BMP4) mRNA. This mechanism results in a rise in BMP4 protein expression levels, which then inhibits downstream Hippo signaling pathways and activates critical effector molecules [321].

Furthermore, m6A regulators are also implicated in cervical cancer-related mechanisms through ncRNAs. HPV16 E7 gene encodes virus-derived circRNA (circE7) that generates the E7 oncoprotein and is linked to cervical cancer. Related research has indicated that the development and progression of CC may be influenced by the mutual regulation of circE7 and m6A modifications [322]. An investigation accomplished by Liang et al. revealed that the demethylase ALKBH5 was capable of removing the m6A modification of circCCDC134 and improving its stability. Subsequently, circCCDC134 acted carcinogenic in CC by sponge-like adsorbing miR-503-5p and stimulating the expression of hypoxia-inducible factor 1a (HIF-1α) [323]. Huang et al. reported that the m6A modification on precursor miR-193b could facilitate its processing into mature miR-193b through METTL3. Further down-regulation of miR-193b encourages the proliferation and invasion of cervical cancer cells by targeting and promoting CCND1-encoding cyclin D1 (CyclinD1) mRNA expression [324].

YTHDF2 and ALKBH5 interact with the lncRNA Growth Arrest-Specific 5 Antisense 1 (GAS5-AS1) to improve the stability of the tumor suppressor gene Growth Arrest-Specific 5 (GAS5) RNA through a m6A-dependent mechanism, thereby delaying CC invasion and metastasis [325]. By modulating the stability and enhancing the biological function of LRRC75A-AS1, IGF2BP1 contributes to the progression of CC by suppressing the ubiquitinated degradation of the NLR family pyrin domain 3 (NLRP3) inflammasome, which is carried out by the ubiquitin ligase SYVN1 [326]. In addition, FTO mediates the m6A methylation modification of lncRNA HOXC13-AS and enhances its stability, thereby upregulating frizzled protein class receptor 6 (FZD6) and further activating Wnt/β-catenin signaling. This process results in the EMT and invasion of CC [327]. Other studies have also detailed the different processes through which m6A modification is implicated in the modulation of CC [325, 328, 329].

In general, m6A modification takes part in cervical cancer through multiple pathways, but the specific mechanism of action needs further exploration.

### m6A in ovarian cancer

OC is a gynecological tumor that is high in invasiveness and mortality. OC often presents with non-specific first symptoms, most cases are realized at an advanced stage, and about 70% of cases recurrent at a later stage [330, 331].

Ovarian carcinogenesis is facilitated by m6A regulators through a range of mechanisms. METTL3 expression levels in ovarian cancer displayed an up-regulation trend [332]. A study discovered that the down-regulation of METTL3 resulted in changes in m6A modification levels, which in turn regulated the stability and translation of oncogene protein kinase B (AKT) mRNA, reduced the phosphorylation activity of AKT protein, and inhibited the progression of OC by down-regulating downstream target cyclin D 1 (CCND1) expression levels [17]. METTL3 could also facilitate the maturation of pri-miR-126-5p and mediate its m6A modification. As a result, miR-126-5p targets and upregulates downstream tensin homolog PTEN expression levels, thereby activating the PI3K/Akt/mTOR signaling pathway and contributing to the progression of OC [18]. By maintaining the stability of estigial Like Family Member 1 (VGLL1) and upregulating its expression levels through the m6A system, METTL3 and IGF2BP2 further activate the VGLL1/High Mobility Group AT-hook 1 (HMGA1)/β-catenin axis, resulting in OC metastasis [333].

The impacts of METTL14-associated m6A regulatory mechanisms on OC is complex and may exhibit both pro- and tumor suppressor potential, dependent upon the specific molecular and cellular context [334, 335]. The significant role of m6A-dependent related mechanisms mediated by WTAP, RBM15, KIAA1429, and HNRNP in the development and progression of OC has been confirmed by numerous studies [163, 336–338]. A study by Liu et al. found that YTHDF1 is responsible for the m6A modification of EIF3C mRNA, which increases its translational efficacy and contributes to the progression of OC [339]. YTHDF2 contributes to the progression of OC by mediating the m6A modification of BMF mRNA and promoting its degradation, thus affecting the process of apoptosis [340]. Conversely, YTHDC1, an additional YTH family reading protein, functions as an OC suppressor. The precise mechanism of action is primarily regulated by the PIK3R1/STAT3/GANAB axis [341].

A study accomplished by Wang et al. on ovarian cancer suggested that IGF2BPs have the capacity to bind the m6A modification on circNFIX, thereby increasing its stability. The overexpression of circNFIX is implicated in tumor malignancy by up-regulating the expression of the miR-647 target gene interleukin-6 receptor (IL-6R) and activating the downstream JAK1/STAT3 signaling pathway [342]. Meanwhile, this process regulates the programmed death ligand 1 (PD-L1) expression to achieve OC-associated immune escape. The TGF-β signaling pathway is believed to be implicated in drug resistance in tumors. One study suggested that the TGF-β pathway may regulate the expression of MDR1 mRNA by modulating the m6A methylation process of RBM15, thereby influencing the chemoresistance of ovarian cancer cells and impacting the prognosis of OC [343]. Additionally, Huang et al. found that FTO-dependent m6A methylation modification is associated with cisplatin resistance in ovarian cancer, although the specific signaling pathways and underlying mechanisms involved in this process remain to be fully elucidated [344].

Furthermore, The expression levels of snail homologous protein 1 (SNAI1) are reduced by FTO in a m6A-IGF2BP2-dependent manner, thereby inhibiting the invasiveness and metastasis of EOC [345]. According to a study conducted by Lv et al., the demethylase ALKBH5 is responsible for the m6A demethylation of lncRMRP, which in turn promotes the expression level of RMRP and facilitates the development of OC [346]. FTO and ALKBH5 demethylation may affect the expression of genes associated with autophagy in OC and contribute to tumor proliferation and invasion [172, 347].

Follicle-stimulating hormone (FSH) has been demonstrated to be involved in the promotion of EMT in OC during specific instances and is associated with the malignancy of OC. An investigation accomplished by Xu et al. investigated the precise mechanism of this theory. FSH increased the expression of ALKBH5, which removed the m6A modification from Snail mRNA. This resulted in higher Snail expression in a m6A-dependent manner. The process led to reduced expression of E-cadherin and promoted mesenchymal genes like N-cadherin, which contributed to OC metastasis [348].

Multiple mechanisms of signaling involved in the development of OC are regulated by the mentioned multiple m6A regulators in a m6A-dependent manner. Nevertheless, there are significant unresolved questions regarding the role of m6A modification in OC.

### m6A in endometrial cancer

EC is an imminent threat to the reproductive health of women, as it is prevalent among Perimenopause and postmenopausal women [349]. Within the past few years, its prevalence has increased.

m6A modification plays a critical role in EC. Liu et al. proposed in a study on EC that METTL3-mediated m6A modification revealed a propensity to be down-regulated, which in turn facilitated the progression of EC by stimulating the AKT pathway and regulating the expression levels of downstream target proteins [350]. The aforementioned mechanisms that drive the malignant effects of EC are also affected by METTL14 mutations. Nevertheless, two additional studies reported conflicting results [351, 352]. Due to this, the biological function of METTL3 in EC remains uncertain.

YTHDF2 and YBX2 have the capacity to collaborate on heat shock protein family A (Hsp70) member 6 (HSPA6) mRNA to regulate HSPA6 expression by modulating its stability, which regulates the survival and progression of cancer cells [353]. Additionally, research by Hong et al. has verified that YTHDF2 decreases EC invasiveness by targeting and downregulating the expression of insulin receptor substrate 1 (IRS1) via the m6A system [354]. The recognition and binding of m6A-modified paternally expressed gene 10 (PEG10) mRNA by IGF2BP1 promotes the expression of PEG10 protein by increasing its stability, which promotes the progression of EC [355]. Numerous investigations confirmed the precise mechanism by which IGF2BP family proteins induce EC invasion [356–358]. Wang et al. proposed that WTAP and IGF2BP3 may collaboratively regulate the expression of the downstream early growth response factor-1 (EGR1)/PTEN axis through m6A methylation. This regulatory mechanism plays a crucial role in modulating the characteristics and progression of EC stem cells [359].

Furthermore, the m6A modification of ncRNAs has been identified as a critical factor in the mechanisms of EC development. For instance, circCHD7 attracts the reader protein IGF2BP2 to bind and enhance the stability of platelet-derived growth factor receptor β (PDGFRB) mRNA through m6A modification. This process further activates the downstream JAK/STAT signaling pathway by upregulating PDGFRB expression and affecting EC progression [360]. In a study of endometrial cancer investigated by Wang et al., it was discovered that LINC00958 attaches to IGF2BP3, which in turn promotes the stability of E2F3 mRNA and boosts the expression of E2F3 gene levels through a m6A methylation mechanism. Consequently, it promotes the invasiveness of EC [361].

FTO activates the Wnt signaling pathway, which in turn leads to EC invasion, by inhibiting HOXB13 mRNA degradation via a m6A modification-dependent pathway [362]. ALKBH5, another demethylase, further facilitates the activation of the insulin-like growth factor 1 receptor (IGF1R) signaling pathway by improving the stability and translational efficiency of IGF1R mRNA by targeting m6A modification [363]. This mechanism results in the proliferation and invasion of EC. In addition, ALKBH5 is a factor which regulates the expression of tumor stemness genes(e.g., SOX2) and impacts the pathogenicity of EC [250].

The invasiveness of EC is influenced by a variety of m6A regulators, which regulate and target the expression of key genes and the activity of signaling pathways. In the future, more studies will identify the rigorous mechanism of m6A modification in the advancement of EC.

### m6A in choriocarcinoma

CCA is a rare and highly aggressive malignant tumor that arises from prenatal trophoblastic tissue. It has a very low occurrence rate but carries a nearly 100% mortality rate unless specific chemotherapeutic drugs are used [364].

The field of m6A modification in CCA is undergoing accelerated development. The most of current investigations on the association between CCA and m6A modification involve mechanisms related to ncRNAs. Lu et al. conducted a study which demonstrated that miR-373-3p, an EMT inhibitor, inhibited CCA invasion by downregulating the TGFβ signaling pathway [365]. MiR935 has been reported to be meaningfully upregulated in CCA tissues [366]. Additionally, a study discovered that METTL3 induces the maturation of miR-935 and mediates its m6A modification, thereby increasing its expression level. Following this, miR-935 targets and inhibits the production of the gap junction protein GJA1, which causes CCA cells to proliferate and invade [367]. Also, Ye et al. confirmed that METTL3 enhances the level of miR-21-5p through the m6A mechanism, which activates the hypoxia-inducible factor asparagine hydroxylase (HIF1A) and further up-regulates the expression of pro-angiogenic factor (VEGF). This increases the blood supply to tumor cells, thereby driving CCA growth and metastasis [368].

Studies on how CCA interacts with other m6A regulators are not available at this time. In the future, we anticipate that additional research will reveal the comprehensive mechanism of m6A modification in CCA, thereby generating novel concepts for the diagnosis and treatment of this kind of disease.

### m6A in adenomyosis and endometriosis

Adenomyosis is a common gynecological condition which is characterized by the invasion of the myometrium by endometrial glands and stroma, resulting in myometrial hypertrophy. Dysmenorrhea and irregular vaginal hemorrhage are the primary characteristics of the disease, greatly affect the quality of life of those impacted [369]. According to published research, m6A regulators may regulate immune responses and cell adhesion during the development of adenomyosis [370]. While there is a scarcity of research on m6A modification in adenomyosis, there is some sign that it may be influential in the endometrium and associated diseases. Zhai et al. confirmed that the METTL3-mediated m6A modification was capable of targeting and regulating the expression of critical genes, including insulin-like growth factor 1 (IGF1) and the detoxifying enzyme DDT, that influenced the invasiveness of adenomyosis. This was achieved by analyzing endometrium-associated gene expression profiles in patients with adenomyosis in the GEO database [370].

EMT is a multipart condition that is initiated by abnormal growth-related mechanisms in the endometrium, some of which are presently unknown [371]. According to a study accompanied by Li et al., bioinformatics analysis indicated that six m6A regulators (METTL3, YTHDF2, YTHDF3, FTO, HNRNPC, and HNRNPA2B1) were differentially expressed among ectopic endometrium and controls. Among them, the growth and invasion of ectopic endometrial cells may be regulated by changes in the expression of HNRNPA2B1 and HNRNPC, which may influence immune mechanisms in endometriosis [372]. Furthermore, another study showed that the down-regulation of methylase METTL3 further impacted DGCR8-mediated splicing processes by reducing the m6A modification level of pri-miR126. This decrease in m6A modification level led to a decrease in the generation of mature miRNA126, which consequently facilitated the migration and invasion of endometriosis [373].

The aforementioned studies offer preliminary insights into the investigation of m6A modification and the correlation between endometriosis and adenomyosis. To cultivate a thorough comprehension of the precise mechanisms of the interaction between the two, additional in vitro verification in the future is required.

### m6A in POI

POI may be influenced by the activity of m6A regulators in regulating germ cell metabolism and function. Cao et al. discovered that the secretion of interleukin 1β (IL-1β) by theca cells was upregulated by decreased levels of m6A modification, which in turn promoted the development of POI by knocking down METTL3 [374]. Pathogenic variants in the reading protein YTHDC2 may influence the normal progression of the meiotic process and contribute to the development of POI [375]. FTO-mediated m6A demethylation has the potential to improve the stability of circBRCA1, which subsequently binds miR-642a-5p and exerts a sponge-like effect. This prevents the interaction of circBRCA1 with the transcription factor (FOXO1) and contributes to the prevention of POI [376]. This conclusion was substantiated by Ding et al. [377].

Furthermore, it has been demonstrated that chemical toxicants, including cyclophosphamide (CTX) and 4-vinylcyclohexene diepoxide (VCD), can impair ovarian function and induce POI. In a rat model of VCD-induced ovarian dysfunction, ALKBH5 acts on YAP mRNA in a m6A-dependent mechanism to further upregulate YAP expression and affect ovarian function [378]. In a human granulosa cell model and a mouse model of POI, investigators discovered that the m6A modification levels in cells or tissues increased substantially in a concentration-dependent manner after CTX treatment, thereby driving the progression of POI [379]. The direction and focus of future research are the specific mechanisms or relevant targets implicated in m6A affecting POI.

### m6A in PCOS

Hyperandrogenism, insulin resistance, and ovulatory dysfunction are frequently encountered in PCOS [380]. The pathogenesis of PCOS is significantly influenced by the m6A methylation modification of mRNA. It has been demonstrated that the activity and protein of insulin-related signal transduction pathways, such as the PI3K/AKT pathway, appear to influence the pathogenesis of PCOS in murine PCOS models through the m6A mechanism [15]. Nevertheless, specific studies are still in the preliminary phases of development.

In addition, various studies have previously shown a correlation between FTO gene abnormalities and the mechanisms that lead to the development of PCOS [381, 382]. FTO expression is positively correlated with androgen levels, and androgen excess is one of the significant manifestations of PCOS. Xue et al. accomplished a study that verified the precise mechanism of FTO-mediated m6A demethylation modification in PCOS-induced androgen excess. Through the m6A mechanism, FTO enhances the stability of key enzymes involved in androgen synthesis, including CYP17A1 and HSD3B2, and up-regulates their expression levels, thereby contributing to androgen synthesis. The inhibition of FTO contributes to a decrease in the expression of these enzymes and the androgen receptor (AR), which in turn reduces the AR/AKT signaling activity and effectively reduces androgen production and secretion, thereby improving the hyperandrogenic state [383]. Another study suggested that FTO could also contribute to insulin resistance in PCOS by removing the m6A modification from the membrane-associated protein FLOT2 (Flotillin-2) mRNA. This would reduce its degradation, increase its stability, and up-regulate FLOT2 expression levels [384].

Moreover, a study of m6A modification in luteinized granulosa cells (GC) from PCOS patients found that YTHDF2 mediated m6A modification of Forkhead Box O3 (FOXO3) mRNA and participated in its degradation in GC from controls group. However, this mechanism was absent in PCOS group, indicating that there is dysregulation of FOXO3 transcription by m6A regulators in PCOS, which may affect pathological development of PCOS [385]. In the future, these discoveries will offer novel concepts for elucidating the fundamental mechanism of PCOS pathogenesis.

### m6A in spontaneous abortion

SA is a frequent complication of pregnancy, and its mechanism is intricate. Numerous researches have verified the association between m6A methylation levels and miscarriage [386, 387]. Huang et al. hypothesized that the m6A modification of Zinc Finger and BTB Domain Containing 4 (ZBTB4) mRNA by METTL3 may be implicated in recurrent miscarriage-associated mechanisms by regulating ZBTB4 expression [388]. Another investigation determined that the reading protein HnRNPC regulates the expression of 5-methyltetrahydrofolate homocysteine methyltransferase (MTHFR) through the m6A mechanism and may induce recurrent miscarriage [389].

The correlation between abortion and benzene exposure was progressively confirmed. Benzo (a) pyrene-7,8-dihydrodiol-9,10-epoxy (BPDE) is a group of chemicals that have reproductive toxicity. They can impact trophoblast function through a variety of pathways, which may result in abortion. According to a study conducted by Xu et al., the methylase METTL14 was upregulated by BPDE exposure. Thus, increased its stability and promoted its expression level by adding m6A modification to lnc-HZ01 and MXD1 mRNA. Consequently, a positive self-feedback loop mechanism was established between the two, which hindered trophoblast proliferation and resulted in abortion [390]. In addition, two additional studies have shown that the up-regulation of lnc-HZ14 [230] and lncRNA-HZ09 [391] is a result of the up-regulation of BPDE exposure, respectively. These mechanisms are related to abortion pathogenesis.

Multiple mechanisms are employed by the demethylase ALKBH5 to induce abortion-associated diseases. Li et al. hypothesis that ALKBH5 could impede trophoblast invasion by increasing the stability of CYR61 mRNA and mediating m6A modification [231]. Another study, which utilized bioinformatics analysis, indicates that the dysregulation of the VEGF signaling pathway may result from ALKBH5 overexpression. This dysregulation impacts the function of macrophages in the endometrium and promotes recurrent spontaneous abortion [392]. In contrast, Zheng et al. discovered that ALKBH5 is capable of up-regulating the expression levels of transcription factors SMAD1 and SMAD5 through the m6A demethylation mechanism, thereby promoting trophoblast viability and preventing spontaneous abortion [393].

Abnormalities of the maternal-fetal interface are frequently linked to spontaneous abortion. FTO-mediated m6A modification processes have been reported to be crucial for the preservation of normal maternal-fetal interfaces. The downregulation of FTO has the potential to result in maternal-fetal-related morbidity, including spontaneous abortion [394]. In research concerning the pathogenesis of unexplained abortion, investigators have also discovered that the FTO-related m6A modification mechanism regulates the MEG3-TGF-β signaling pathway, thereby influencing chorionic invasiveness and inducing abortion [395].

Investigating the relationship between the mechanism of abortion and m6A will aid in comprehending the molecular underpinnings of abortion and may yield novel clinical preventive and therapeutic strategies.

### m6A in preeclampsia

As previously mentioned, the mechanisms by which m6A regulators regulate the invasion and migration of placental trophoblasts are involved. However, the progression of PE may be influenced by aberrant trophoblastic invasion. Related research has shown a strong correlation between the development of PE and the upregulation of m6A modification levels [208, 396]. In contrast, there are also indications that the levels of m6A modification are downregulated in PE. Complex mechanisms associated with the METTL3-myosin light chain kinase (MYLK) axis and the WTAP/IGF2BP1/high mobility group nucleosome binding domain 3 (HMGN3) axis are implicated in this discovery [228, 397]. Further investigation of its impact on m6A modification levels will assist in the identification of the pathogenesis of PE.

In a study conducted by Chen, it was suggested that METTL3 and YTHDF2 reduce the level of transmembrane BAX inhibitor 6 (TMBIM6) by regulating the m6A modification of TMBIM6 mRNA and promoting its degradation. This process results in the dysfunction of trophoblasts and the development of PE [398]. Newly published research has also discovered that METTL3 is implicated in the progress of PE by influencing the iron apoptotic mechanism of trophoblasts. It regulates the m6A modification of Acyl-CoA Synthase Long Chain Family Member 4 (ACSL4) mRNA through a specific mechanism [399].

Furthermore, in HTR-8/SVneo cells, the up-regulation of METTL14 alters the stability and translation of FOXO3a mRNA by increasing m6A modification. This results in increased FOXO3a expression, which in turn leads to PE development [400]. One study discovered that the overexpression of the methylase RBM15 reduced CD82 expression and facilitated trophoblastic invasion and PE development by increasing the levels of m6A modification, promoting YTHDF2 recognition and binding to CD82 mRNA, and enhancing its degradation [401]. In contrast, ALKBH5 has the potential to alleviate the pathological process of PE by increasing the expression level of PLAC8 by eliminating the m6A modification on placenta-specific 8 (PLAC8) mRNA [402].

The ncRNAs are also regulated by the m6A regulator to influence trophoblast function in PE through a variety of mechanisms. Wang et al. confirmed that METTL3 mediates the m6A modification of lncRNA HOXD-AS1 and maintains its stability. Subsequently, the up-regulated HOXD-AS1 recruits miR-135a and exerts a sponge-like effect, relieving its inhibition of the target gene anti-apoptotic protein β-repein transducat-containing protein (β-TRCP) mRNA. This contributes to the upregulation of β-TRCP expression, stimulation of the NF-κB signaling pathway, and an impact on PE development [403]. Similarly, the circSETD2/miR-181a-5p/Myeloid Cell Leukemia 1 (MCL1) axis, which is mediated by METTL3, is also implicated in the development of PE [404].

METTL14 and IGF2BP3 may also be responsible for the regulation of circPAPPA2 m6A methylation stability and the enhancement of its expression, which can impact the progression of PE and placental function [405]. Finally, the regulation of angiogenesis-related gene expression in trophoblasts may be facilitated by the IGF2BP2-mediated m6A modification of linc01116 RNA, which offers a novel perspective on the development of PE [406].

### m6A in GDM

The manipulation of insulin sensitivity, inflammatory response, placental function, and cellular metabolism is the fundamental mechanism of m6A modification in GDM [407–409]. One study suggests that METTL3-dependent m6A modification enhances the stability of hsa_circ_0072380, potentially influencing the pathogenesis of GDM by regulating pathways or gene expression associated with these factors [410]. Furthermore, Ning et al. elucidated the pivotal role of m6A modification in placental development in GDM, demonstrating that FTO regulates the fusion process of trophoblast cells by modulating the stability and expression of SIK1 mRNA [411]. A study that utilized GEO data indicated that m6A-modified LINC00667 may be involved in the development of GDM by modulating the expression of downstream GDM-related target genes in response to the recognition and binding of YTHDF3 [412]. This conclusion must, however, be verified in in vivo or in vitro models.

In high glucose-cultured HTR8/SVneo cells, METTL14 inhibits FOXO1 expression, enhances trophoblast function, increases downstream miR-497-5p expression levels, and mediates XIST silencing through the m6A mechanism, thereby impeding the progression of GDM [413]. Chen et al. discovered that chemokine ligand 5 (CCL5) levels were generally higher in GDM patients. Conversely, METTL14 possibly influenced the progression of GDM by mediating m6A modification of CCL5 and affecting its stability, which may have resulted in the promotion of proliferation, migration, and apoptosis of trophoblasts [414]. In addition, Fang et al. discovered that RBM15 regulates CLDN4 expression through m6A modification, which subsequently affects hepatic glucose and lipid metabolism in the progeny of GDM mice, resulting in insulin resistance [415].

Currently, there are insufficient studies on the function of m6A modification in GDM regulation, and the precise mechanism is unclear. Therefore, further studies are necessary. Future research should aim to validate these preliminary findings through more sufficient in vivo and in vitro experiments, alongside large-scale, multicenter clinical studies, to further elucidate the biological functions and underlying mechanisms of m6A modification in GDM. Such studies will provide a more robust and reliable foundation for a comprehensive understanding of the role of m6A modification in the pathogenesis and progression of GDM.

## Treatment and prognosis

The m6A modification mechanism in various of gynecological tumors has been progressively elucidated, which is anticipated to promote the creation of innovative therapeutic strategies and enhance the prognosis of patients. Furthermore, the potential of m6A modifiers as biomarkers for the early diagnosis and prognosis of cancer is increasingly recognized and investigated. While traditional tumor markers (e.g., CA125, HE4,SCC) and diagnostic methods (e.g., HPV detection, TCT) have played an important role in the early detection and monitoring of gynecological cancers, their clinical applicability is constrained by limitations such as low sensitivity, poor specificity, and insufficient capacity for dynamic monitoring. Future advancements in quantifying and localizing specific m6A modification levels and loci within blood exosomal RNA and tissue mRNA hold promise for overcoming these challenges. Such approaches could provide powerful tools for the early and accurate diagnosis, biomarker discovery, and the personalization of treatment strategies for gynecological malignancies. However, the clinical application of m6A modification in early detection remains largely in the basic research phase, with no existing clinical studies to date. Thus, further standardization and rigorous clinical validation will be necessary to translate these findings into clinical practice.

In CC, m6A methylation levels are significantly correlated with cancer progression and survival. The levels of ZC3H13, YTHDF1, and YTHDC1 expression in cervical squamous cell carcinoma (CESC) were found to significantly correlate with the prognosis of CESC by bioinformatics analysis [416]. These proteins may also be useful targets for cancer therapy. According to Wang et al., the aforementioned conclusions were partially corroborated by the findings of a biological analysis study of CC patients in the GEO and TCGA databases. The study revealed that RBM15 is a prognostic biomarker in CC, and ZC3H13 may impact overall survival (OS) in CC patients [417]. Therefore, further mechanistic investigations are necessary to validate these conclusions in the future.

A study confirmed that YTHDF1 induces the expression of RANBP2 (RAN-binding protein 2) through a m6A-dependent mechanism, thereby facilitating CC metastasis [19]. The m6A modification of low-density lipoprotein receptor-related protein 6 (LRP6) mRNA is mediated by YTHDF3, which subsequently promotes the expression of LRP6 protein and activates downstream signaling pathways. This process is responsible for the invasion of CC and the metastasis of lymph nodes [418]. YTHDFs proteins may have a significant impact on CC treatment, as suggested by relevant studies.

A study on the mechanism of CESC resistance revealed that FTO, through eliminating m6A alteration on β-catenin mRNA, increasing its stability, and up-regulating expression levels, might boost patient chemoresistance and lessen the lethal effect of chemotherapeutic medicines on cancer cells [419]. Centromere protein K (CENPK) is also a contributing factor to recurrence and drug resistance in CC. A study discovered that the Wnt/β-catenin signaling pathway was activated by methylase ZC3H13, which increased the level of CENPK mRNA and mediated the m6A methylation modification process. This process resulted in the progression of CC and development of chemoresistance [420]. In cancer management, early detection of potential recurrence, assessment of patient response to treatment, and prediction of prognosis are crucial for optimizing therapeutic strategies and improving patient survival and quality of life. Therefore, dynamic monitoring of m6A modifier expression levels, coupled with the development of predictive models based on these markers, could provide a more comprehensive evaluation of treatment responses, recurrence risks, and long-term prognoses. Additionally, clinicians could stratify patients into high-risk and low-risk groups based on m6A modification factor expression, which would offer a foundation for personalized treatment plans. Currently, most in vivo and in vitro studies primarily focus on the expression levels of m6A modification-related molecules and their correlation with patient prognosis. However, further large-scale cohort studies are required to validate these findings, ensuring a robust clinical translation of m6A methylation modifications in reproductive system diseases.

The results of numerous bioinformatics-related researches have indicated that m6A modifier regulators have significant prognostic potential in OC [421–428]. A study based on OC patients in the TCGA database revealed that high expression of WTAP, YTHDF1/2/3, FTO, and ALKBH1/5, while low expression level of METTL14 may be associated with poor prognosis in OC [425]. Li et al.‘s study indicates that VIRMA, IGF2BP1, and HNRNPA2B1 may be correlated to the prognosis of OC. The risk prediction model has been found to be effective based on the aforementioned conclusions. In addition, the investigators employed the miRbase database to screen upstream miRNA targets of the aforementioned genes and discovered that miR-196b-5p may target IGF2BP1, modulate the expression level of downstream PTEN protein, and lead to OC progression [422]. Zhu et al. additionally demonstrated the predictive role of KIAA1429 and YTHDC2 in OC [426]. Nevertheless, these findings are only suitable for use as a preliminary reference, as they are not supported by confirmatory in vitro experiments.

Yu et al. conducted an experimental study on high-grade serous ovarian cancer (HGSOC) that confirmed elevated WTAP expression as a poor predictor of HGSOC [429]. This research partially corroborates the conclusions of Han et al. [425]. Sun et al. also discovered that ALKBH5 overexpression may be involved in pertinent mechanisms that mediate OC lymph node metastasis [430]. A study conducted by Ye et al. was innovative in that it combined machine learning algorithms to screen m6A modification-related lncRNAs in serous ovarian cancer (SOC) and establish a predictive model (m6A-LRM). The study found that METTL3 down-regulation may be involved in regulating RP11-508M8.1 (one of the selected lncRNAs) and promoting OC cell invasion [428]. The model demonstrated a high degree of predictive accuracy for the treatment effect of SOC and OS in the subsequent validation trial.

Platinum medications serve as the foundation of initial OC treatment. A study on cisplatin resistance in OC patients discovered that METTL3 increased the stability of lncRNA RHPN1 antisense RNA 1 (lncRNA RHPN1-AS1) in a m6A-dependent mechanism. This, in turn, resulted in increased cisplatin resistance in OC patients by upregulating the downstream PI3K/AKT pathway [431]. Another study also demonstrated that the METTL3/YTHDF2 axis also downregulates interferon-inducible RNA helicase 1 (IFFO1) expression by mediating the m6A modification of IFFO1 mRNA and promoting its degradation, leading to cisplatin resistance and metastasis in ovarian cancer [432].

Furthermore, Yuan et al. verified that the RBM15 expression level can predict paclitaxel resistance in OC patients. The specific mechanism is affected by the transforming growth factor-β (TGF-β)/RBM15/multidrug resistance protein 1 (MDR1) axis, in which RBM15 regulates MDR1 expression through the m6A mechanism, thereby affecting the effect of chemotherapy [343]. Specific mechanisms of ALKBH5 [433], FTO [434], and YTHDF1 [248, 435] in OC cisplatin resistance, as well as METTL3 [436] in OC carboplatin resistance, have also been reported in numerous studies.

Given the m6A regulators previously mentioned, there is an increasing interest in the investigation of targeted agents that are linked to OC resistance. For instance, AE-848 has the capacity to function as a targeted agent for OC therapy by destabilizing IGF2BP3-targeted mRNA, thereby impeding the growth and progression of OC cells [437]. Sulfonamides (Sul) are potential targets for OC therapy and may influence the process of apoptosis through the METTL3-mediated m6A mechanism [438]. As prospective biomarkers and therapeutic targets in OC, FTO and ALKBH5 may be implicated in PARP inhibitor (PARPi)-related biological mechanisms [439]. However, research on targeted therapies related to m6A modification has yet to reach the clinical trial stage. Furthermore, most existing studies rely on animal and cellular models, which do not adequately reflect the complexity of human tumors, particularly in terms of the tumor microenvironment, immune response, and treatment response. The intricate nature of m6A modification mechanisms, combined with tumor heterogeneity and individual variability, makes it challenging for current models to fully capture the dynamics of tumor growth and metastasis. Moreover, existing studies lack an in-depth exploration of long-term tumor progression and the mechanisms underlying drug resistance, which significantly restricts the clinical translation and application of m6A modification research findings. Future research should concentrate on this topic in order to elucidate the molecular mechanisms which may aid in the development of clinical treatments.

The regulatory mechanisms of pertinent prognostic biomarkers in EC are also influenced by the m6A modification and its regulators. Based on CpG sites in the m6A regulator, specific prognostic prediction models have been developed in related studies that are effective in foreseeing the prognosis of patients with EC [440]. Zhai et al. hypothesized that FTO, YTHDF1 and RBM15 are ‌important‌ factors in the prognosis of EC patients in a study that utilized the TCGA dataset of EC patients [205]. An additional experimental study conducted recently also verified that the elevated METTL3, METTL14, FTO, HNRNPC, and HNRNPA2B1 expression levels are associated with a shorter OS in EC patient [351]. In addition, Zhan et al. discovered that METTL3 and YTHDF2 are involved in the m6A modification process of NLRC5 mRNA, which upregulates its stability and inhibits degradation. This results in an increase in NLRC5 expression levels, a reduction in NLRC5-mediated immune surveillance, and a participation in EC progression [441]. The IGF2BP1 is involved in the process of EC cell metastasis, as it binds to and promotes the expression and accumulation of TRKB protein in a m6A-dependent manner [358].

Moreover, two studies accomplished by Shi [442] et al. and Shan [443] et al. demonstrated a correlation between the expression levels of m6A-related lncRNA and the survival of ECs, as well as immune-related mechanisms. Consequently, it is conceivable that the m6A regulators listed above may be promising targets in the development of EC, and the inhibitors or activators that are generated may have potential regulatory effects on the treatment and prognosis of EC patients.

Limited research has been conducted on the topic of EC treatment and resistance. It has been reported that METTL14 and YTHDF2 are indirectly implicated in the progression of iron death by influencing the methylation process of protein arginine methyltransferase (PRMT) through the m6A mechanism. However, the aforementioned processes are impeded by PRMT inhibition, which enhances the sensitivity of endometrial cancer cells to iron death and offers a novel perspective on EC treatment and drug resistance research [444]. In summary, clinicians will be able to detect and treat gynecological malignancies at an early period, thereby enhancing the survival rate of patients, by conducting a thorough analysis of the high-risk factors associated with their occurrence and development.

Additionally, treatment of other female reproductive system diseases and m6A-related mechanisms. In a rat model of PCOS, Zhang et al. found that Cangfu Daotan Decoction (CDD) methylation of m6A decreased the activity of the Wnt/β-catenin pathway, thereby improving the recovery of ovarian function [445]. Another study suggested that butyric acid supplementation could alleviate the symptoms of PCOS by inhibiting the expression of METTL3 and reducing the stability and expression of FOSL2 mRNA. This would down-regulate the expression of NLRP3 and further decrease the expression of downstream inflammatory factors [446].

There are inflammatory factors that contribute to the development of ovarian senescence, whereas melatonin has a potent antioxidant effect. Zhu et al. demonstrated that melatonin mitigates the decline in ovarian function by regulating the expression of the m6A regulator YTHDF2 and reducing its mediated mRNA degradation, further blocking the MAPK-NF-κB signaling pathway and achieves anti-inflammatory objectives [447]. Interesting, it has been shown that melatonin is also essential for the protection of pregnancy by modulating inflammatory and apoptosis-related pathways through the m6A mechanism [448]. Finally, Chen et al. confirmed that m6A expression was significantly elevated in trophoblasts of GDM patients with obesity compared to non-obese individuals. Hesperidin was able to indicate its potential role in the treatment of GDM by participating in the regulation of autophagy and m6A methylation levels [449]. In the future, more comprehensive research is needed to investigate the specific mechanism of m6A modification in the treatment of female reproductive system diseases.

The development of drugs targeting m6A modification regulators represents a cutting-edge frontier in the field of RNA epigenetics. To date, clinical trials of related therapeutics have progressed relatively slowly, with the majority of researches still in the basic research or early-phase clinical trial stages. Currently, m6A-modifying regulators under investigation include METTL3 inhibitors such as STM2457 [450] and STC-15, as well as FTO inhibitors like MO-I-500 and R-2-hydroxyglutaric acid (R-2HG) [451], which have shown potential for treating various solid tumors and related diseases. These compounds are anticipated to emerge as novel therapeutic agents in the RNA epigenetics domain. Notably, a clinical trial (NCT05584111) evaluating the METTL3 inhibitor STC-15 in advanced solid tumors is underway, with preliminary results expected by 2024. With an increasing understanding of the molecular mechanisms underlying m6A modification and the continuous evolution of drug development technologies, it is projected that more m6A-targeted therapies will advance into clinical trials, potentially offering new treatment avenues for obstetric and gynecological diseases.

## Conclusion and future perspectives

The m6A modification is a highly prevalent methylation modification in RNA that is critical for cellular physiology and pathology. In this study, we present a comprehensive overview of m6A-related regulators and their biological function-related mechanisms, the development history of m6A detection methods, the various roles that m6A modification plays in the female reproductive system, and their possible connections to associated diseases.

Additionally, the m6A modification has a multifaceted effect on the development of related maladies and the health of female reproductive systems. It regulates t the maturation and development of reproductive cells, the synthesis and signal transduction of reproductive hormones, the maintenance of maternal-fetal microenvironment homeostasis, and the mechanisms related to embryonic development. This perspective provides obstetricians and gynecologists with a fresh understanding of the diseases in question. Currently, the primary bottlenecks and challenges in the field of m6A modification include the unclear and complex regulatory mechanisms, the experimental model is not fully representative, and the limited accuracy of detection technologies. Although m6A modification has shown potential in the occurrence, progression, and targeted therapy of various diseases, its clinical application as a biomarker remains in the early stages. There is also a lack of m6A-targeted drugs or therapeutic strategies, and numerous technical and biological hurdles must still be overcome for successful clinical translation. The gap between basic research and clinical application is hindered by both technical and practical obstacles. To address these challenges, it is essential for basic research to focus on standardizing m6A detection methods and improving their accuracy. Additionally, collaboration with clinical practice should be strengthened through large-scale, multi-center studies to validate findings and facilitate the integration of m6A modification research into clinical settings. Moreover, future research should explore the interactions between m6A modification and other epigenetic modifications, such as DNA methylation and histone modifications, particularly in gynecological cancers. Investigating how these epigenetic changes synergistically or mutually regulate gene expression and cellular function will provide deeper insights into the mechanisms driving disease initiation and progression, thereby offering a more comprehensive theoretical foundation for early diagnosis, prognosis evaluation, and targeted therapies.

This study has several limitations. Many of the studies related to m6A modification and obstetric and gynecological diseases included in this review have small sample sizes and primarily focus on cell lines and small animal models, which are not supported by large-scale clinical data. While these experimental models provide essential insights into the biological mechanisms of m6A modification, they may not fully capture the complexity and diversity of human diseases. Additionally, small sample studies are prone to statistical biases that could affect the reliability of the findings. Therefore, future research should prioritize the collection of clinical samples, particularly through multi-center, large-scale clinical cohort studies, to more comprehensively assess the clinical relevance of m6A modification in the onset, progression, and prognosis of diseases of the female reproductive system.

In addition, while relevant studies have highlighted the potential role of m6A modification in diseases of the female reproductive system, particularly cancer, the underlying molecular mechanisms and the complex regulatory networks involved remain not fully elucidated. One example is that the scarcity of researches on the regulatory mechanisms of m6A modification on immune cells and reproductive cells in various pathological conditions. Additionally, the regulatory mechanisms of the m6A regulator are complex, as they may serve distinct functions in various diseases. Moreover, the regulatory mechanism of the m6A mechanism in specific gynecological diseases (e.g., PCOS, endometriosis, GDM, etc.) has not been thoroughly examined.

Therefore, future research should focus on elucidating the detailed molecular mechanisms of m6A methylation modification in regulating gene expression, and investigate how these processes influence the beginning and progression of female reproductive system diseases. Additionally, it is crucial to explore strategies for enhancing the specificity and precision of targeted therapies, addressing potential drug resistance issues that may arise during treatment. This will provide theoretical support for the development of individualized treatment plans and facilitate the translation of m6A modification mechanisms into clinical practice, ultimately advancing therapy in clinical settings.

## Data Availability

All data in this review are publicly available.
